# Influence of Solvent Selection on the Crystallizability and Polymorphic Selectivity Associated with the Formation of the “Disappeared” Form I Polymorph of Ritonavir

**DOI:** 10.1021/acs.molpharmaceut.4c00234

**Published:** 2024-06-20

**Authors:** Chang Wang, Cai Y. Ma, Richard S. Hong, Thomas D. Turner, Ian Rosbottom, Ahmad Y. Sheikh, Qiuxiang Yin, Kevin J. Roberts

**Affiliations:** † Centre for the Digital Design of Drug Products, School of Chemical and Process Engineering, University of Leeds, Woodhouse Lane, Leeds LS2 9JT, U.K.; ‡ School of Chemical Engineering and Technology, State Key Laboratory of Chemical Engineering, Tianjin University, Tianjin 300072, China; § Molecular Profiling and Drug Delivery, Research and Development, AbbVie Inc, North Chicago, Illinois 60064, United States

**Keywords:** solvent selection, disappeared polymorphic form, crystallizability, nucleation kinetics, ritonavir, intra- and intermolecular interactions, hydrogen bonding, conformational polymorphism, molecular dynamics

## Abstract

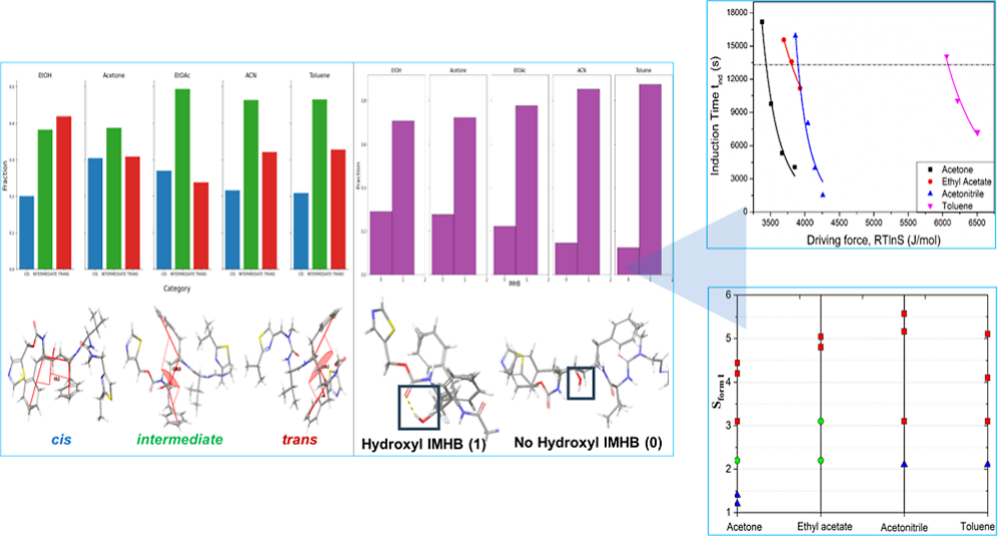

The comparative crystallizability and polymorphic selectivity of ritonavir, a novel protease inhibitor for the treatment of acquired immune-deficiency syndrome, as a function of solvent selection are examined through an integrated and self-consistent experimental and computational molecular modeling study. Recrystallization at high supersaturation by rapid cooling at 283.15 K is found to produce the metastable “disappeared” polymorphic form I from acetone, ethyl acetate, acetonitrile, and toluene solutions in contrast to ethanol which produces the stable form II. Concomitant crystallization of the other known solid forms is not found under these conditions. Isothermal crystallization studies using turbidometric detection based upon classical nucleation theory reveal that, for an equal induction time, the required driving force needed to initiate solution nucleation decreases with solubility in the order of ethanol, acetone, acetonitrile, ethyl acetate, and toluene consistent with the expected desolvation behavior predicted from the calculated solute solvation free energies. Molecular dynamics simulations of the molecular and intermolecular chemistry reveal the presence of conformational interplay between intramolecular and intermolecular interactions within the solution phase. These encompass the solvent-dependent formation of intramolecular O–H...O hydrogen bonding between the hydroxyl and carbamate groups coupled with differing conformations of the hydroxyl’s shielding phenyl groups. These conformational preferences and their relative interaction propensities, as a function of solvent selection, may play a rate-limiting role in the crystallization behavior by not only inhibiting to different degrees the nucleation process but also restricting the assembly of the optimal intermolecular hydrogen bonding network needed for the formation of the stable form II polymorph.

## 1 Introduction

Polymorphism is related to the ability of a compound to crystallize in different crystallographic structures but with the same chemical composition.^1−6^ For organic materials, where the solid-state chemistry is mostly characterized by a combination of weak undirected van der Waals interactions coupled, in some cases, with stronger directed hydrogen bonds (HBs), polymorphism is not an unexpected phenomenon (e.g., ref (7)). Indeed, it has often been reported that the number of polymorphic forms discovered for a given material depends upon the efforts (time and resources) committed.^4,5,8^ In conformational polymorphism, flexible organic molecules can adapt different molecular conformations, resulting in the different polymorphic forms.^3,9,10^ As a result, a multitude of polymorphic forms may be observed e.g., in tolfenamic acid and paritaprevir,^11−25^ ritonavir,^26−34^ ROY,^35,36^ acridine,^37^ and aripiprazole.^38^ For pharmaceutical materials, the different polymorphs of a substance may exhibit different physicochemical properties, such as solubility, stability, density, and bioavailability.^1,39,40^ Therefore, gaining an understanding and then control of the crystallization of an active pharmaceutical ingredient (API) from solution can form an essential part of both its development and subsequent formulation as a drug product. The polymorphic stability of the final product is also important industrially due to the potential impact of any polymorphic transformation upon notably product stability and hence on shelf life and dissolution and hence bioavailability.^27,41^

Crystallization from solution is important for the separation and purification of organic ingredients.^42,43^ Solvent selection has been known to alter polymorphic behavior (e.g., refs (44,45)) but the exact mechanism for this has not, as of yet, been fully characterized. However, the mechanisms could include the effects of solute/solvent interactions in the solution state^46−48^ on the nucleation process and hence on polymorphism.^49,50^ Nucleation can play a significant role in controlling the final product properties^7,51−53^ with its rate being very sensitive to solution chemistry.^16,45,47,54−58^ Using classical nucleation theory (CNT), the interfacial energies between the solid and solution forms can be calculated and correlated to solvent properties such as solubility,^59^ boiling point,^60^ and dipole moment^60^ to examine nucleation behavior.^61−63^ Although, nucleation from solution can be strongly influenced by solvent selection,^64^ the detailed mechanisms underpinning solvent-dependent behavior also remain quite elusive. In this respect, modeling intermolecular solute/solvent interactions provided helpful insights into both the strength of solvation interactions and the associated solvent coordination to solute molecules as a function of solvent type.^11,48,65−68^ Crystallizability as a function of solvent selection has also been assessed through meta-stable zone width (MSZW) measurements^11,69^ and also through the determination of nucleation kinetics using CNT.^46,47,60,70,71^

In silico methods, such as molecular dynamics (MD) simulations, have been widely used to capture the dynamical behavior of the explicit intermolecular interactions within the solution phase both qualitatively and also quantitatively by developing an understanding of the molecular and intermolecular assembly behavior during the crystal nucleation process associated with crystallization from solutions (e.g., refs (43,72–77)). Recently, some detailed methods have been developed to characterize the propensity of incipient bulk synthons within the solution state through the targeted analysis of the MD trajectory files.^74,78,79^

**Figure 1:**
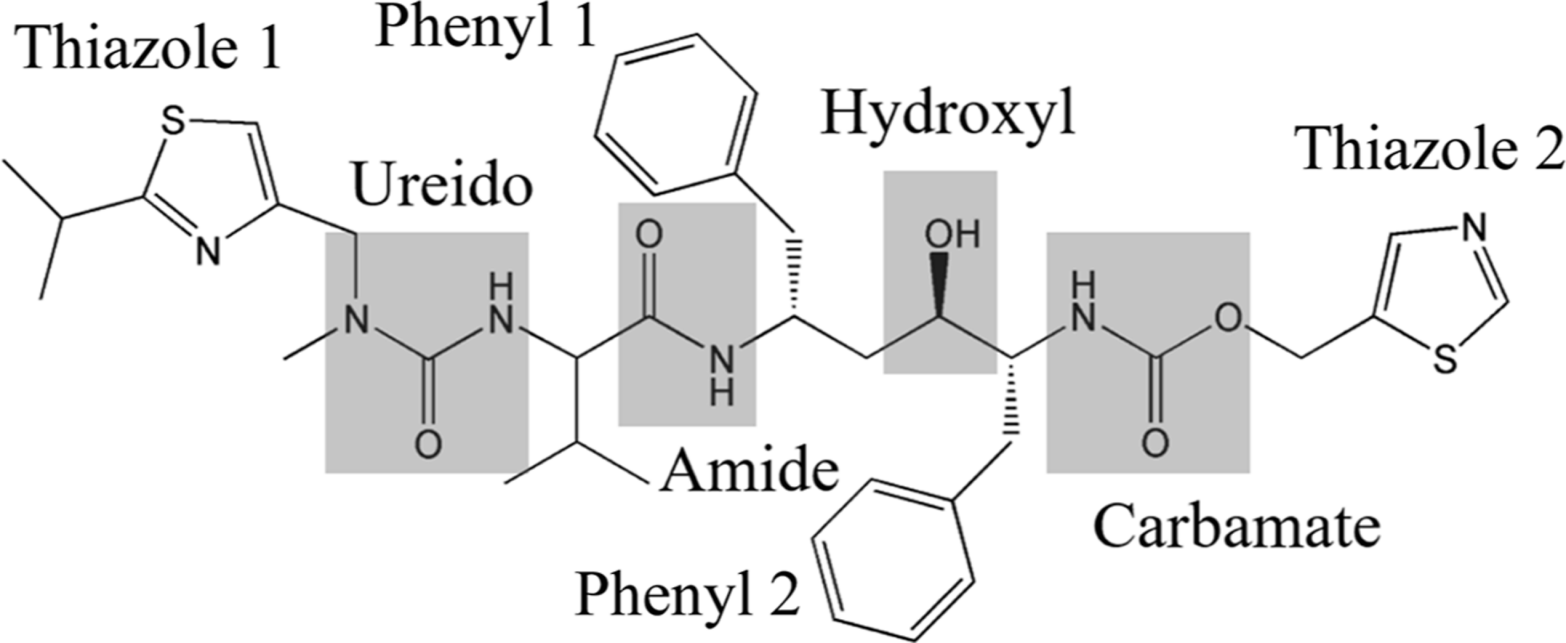
Chemical diagram of the ritonavir molecule highlighting some of the important functional groups.^26,28−30^

Ritonavir (Figure 1) is a highly representative example of a high molecular weight pharmaceutical API. Since Bauer’s seminal paper^26^ published over 20 years ago, there has been significant interest within the medical and pharmaceutical communities in ritonavir with a number of high-impact papers on its solid-state characterization.^28−31,33,80,81^ The drug has also attracted interest in an antivirus medication for Covid-19 treatment.^82^ In the current work, the nucleation behavior of the (now) metastable and “disappeared” form I has been examined using isothermal induction time measurements carried out as a function of solvent selection using apolar, polar aprotic, and polar protic solvents. In this work, the interfacial energy, critical nucleus radii, number of molecules in the critical nucleus, and the attachment frequency of building units to a nucleus have been determined by using CNT together with an evaluation of the possible nucleation mechanism of the metastable form within the different solvents. The nucleation data were rationalized with respect to the atomistic scale through related MD simulations in which the conformational energy landscape of the ritonavir molecule within explicit solvent environments has been used to capture the dynamic interplay between inter- and intramolecular interactions, and their associated conformational preferences. These have been supported by free energy perturbation MD simulations through which the solvation energies for all five solvents, such as ethanol (EtOH), acetone, acetonitrile (ACN), ethyl acetate (EtOAc), and toluene, were calculated.

## 2 Molecular and Crystal Chemistry and Their Interrelationship

In early phase development, ritonavir was characterized to have only one polymorphic form (hereinafter form I).^26^ However, two years after it had been released on the market, a new slowly nucleating and more stable polymorph (form II) with a lower solubility^26^ appeared resulting in all the manufactured material final products failing their dissolution QA tests and making the originally marketed form I a “disappeared polymorph”. Subsequent attempts to continue to manufacture and stabilize form I were reported to have been unsuccessful,^26,27,32,83^ leading to further research on the properties of the new stable form II and its formulatability.^84−86^ Ritonavir is a conformationally flexible molecule, and as such later and more extensive polymorphic screening studies revealed its ability to crystallize in many more different forms with a current landscape encompassing three polymorphs and two solvates, a quaternary solvated cocrystal salt and an amorphous glassy form.^28−31,33,85,87^ Despite these studies, there remains very little available literature data regarding the nucleation behavior of the ritonavir API as a function of supersaturation for a range of organic solvents reflecting, in part, its inherently low crystallizability and high glass forming ability.^88,89^

The structures of form I and II polymorphs were determined and thoroughly characterized by Bauer et al.^26^ Wang et al.^28^ have subsequently characterized and compared, in significant detail, the crystal chemistry for both forms I and II in terms of their molecular conformations, polarizabilities, hydrogen bonding networks, intermolecular packing structures, and crystal morphologies and have cross-correlated these to a wider assessment of their crystallizability behavior and respective surface properties.

Examination of the two crystal structures reveals that the distinct differences in molecular conformation were found to be associated with the carbamate, ureido group, and phenyl conformations (Table 1), with the form I adopting a trans and cis conformation for its carbamate and ureido groups, respectively, whereas form II adopts a cis and trans conformation of the same groups.^26,28,29,83^ Detailed analysis of the gas phase conformational energy landscape^83^ revealed that the form I carbamate group trans conformation was found to be energetically more stable than the form II cis conformation and that there is a significant conformational energy barrier (cis to trans: 13.4 kcal mol^–1^; trans to cis: 16.6 kcal mol^–1^) which effectively inhibits any easy transformation between these two conformational states. The more stable form I conformer was also found to be closer to the previously identified global minimum conformation of ritonavir.^83^ Despite the fact that form I is more closely packed, its hydrogen bonding pattern was found to be less optimal than that in form II,^90^ and therefore solvent selection plays a role in the nucleation and polymorphic transformation process.^44^ Close examination^28^ of the functional groups within the molecular fragments reveals that the different energetic contributions from these functional groups is consistent with the stronger hydrogen bonding in form II. In this, the cis conformation of the carbamate group rotates the adjacent phenyl group away from shielding the hydroxyl group, thus enabling it to act as both a donor (to the ureido group) and acceptor (from the amide group). This is in direct contrast to form I where its access is much more constrained and hence where it can only act as a donor (to the thiazole 2 group). While the trans conformation of the carbamate group enables a higher density and degree of close packing of the phenyl rings in the form I structure, in doing so it restricts opportunities for the hydroxyl group to achieve its optimal hydrogen bonding configuration.

**Table 1:** Summary of the Configurations of the Carbamate, Ureido, and Phenyl Conformations of the Constituent Molecules of the Form I and II Structures, Together with Their Associated HB Networks

polymorphic form	carbamate conformation	ureido conformation	phenyl conformation	HB network	hydroxyl group
I	trans	cis	cis	mostly dimeric stacking	mostly shielded
II	cis	trans	trans	4 HB ring structure	more open

**Figure 2:**
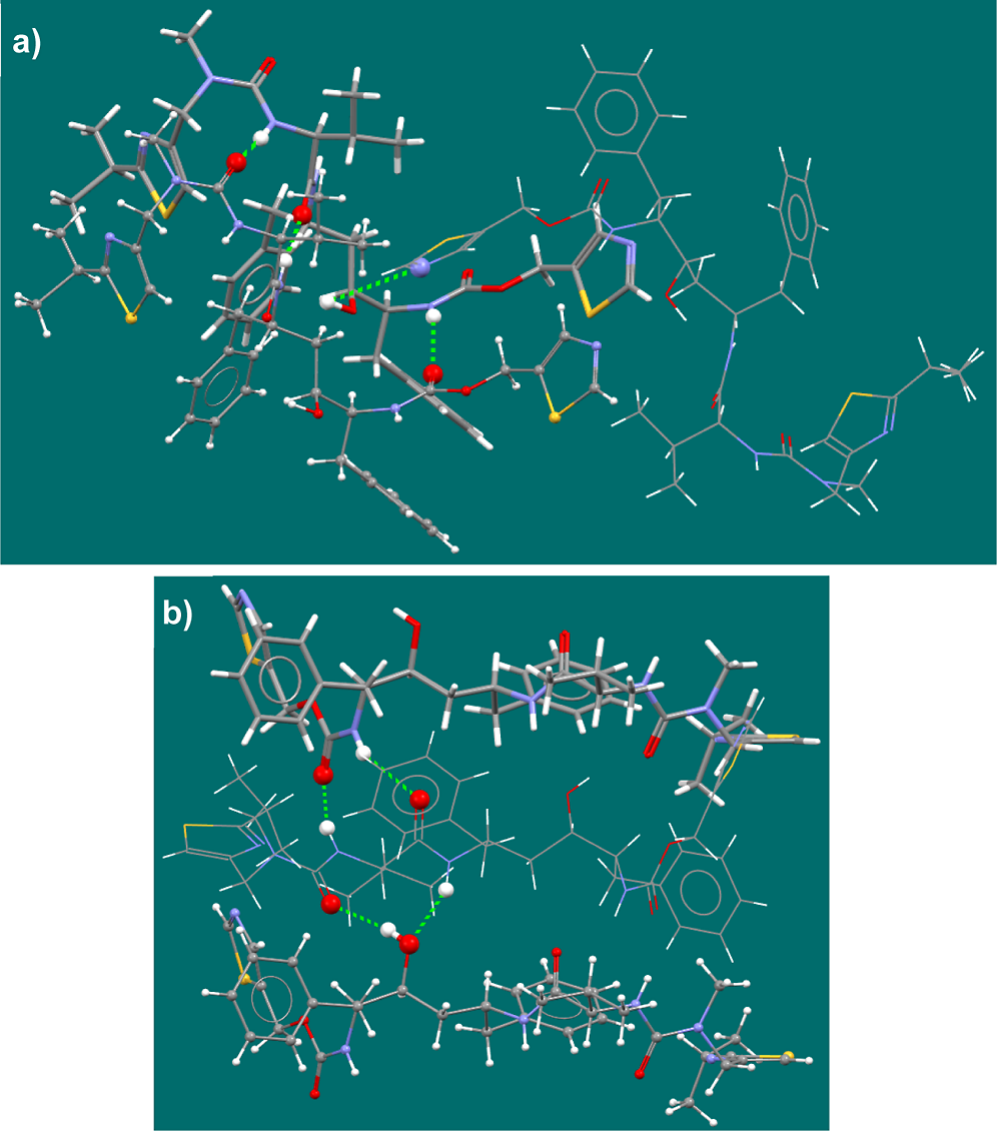
Intermolecular HB networks involving three molecules (wireframe, capped sticks, ball and stick) with HB (green dashed line) atoms highlighted as spacefill: (a) form I with three homo HBs and one hetero HB and (b) form II with four hetero HBs.

Ritonavir has four HB donors involving one hydroxyl O–H and three amidic N–H groups together with twelve acceptors,^28^ leading to the formation of four intermolecular HBs (Figure 2) but without the formation of any intramolecular HBs in the crystal structures of the two forms. The detailed analysis of the HB networks for both forms I and II can be found in the literature (e.g., refs (28,29)) and, as discussed,^26^ a significant distinguishing difference of the HB structure between the two forms is related to the hydroxyl functional group. For form I, two characteristic intermolecular synthons were identified^28,29^ with synthon A_I_ comprising three strong homo intermolecular HBs (Figure 2a).

N–H (amide)...O=(amide);
N–H (ureido)...O=(ureido);
N–H (carbamate)...O=(carbamate),

These 3 HBs form a ″dimeric” closely packed stacking sequence. The second synthon B_I_ has just a single weaker hetero intermolecular HB (Figure 2a):

O–H (hydroxyl)...N (thiazole 2).

In contrast, for form II, a single synthon A_II_ was found^28,29^ comprising four hetero intermolecular HBs (Figure 2b).

N–H (ureido)...O=(carbamate);
N–H (carbamate)...O=(amide);
N–H (amide)...O–(hydroxyl);
O–H (hydroxyl)...O=(ureido),

These 4 HB interactions form a characteristic ring-like network involving the four functional groups (Figure 2b).

Overall, the complex molecular conformational states and intermolecular crystal chemistry for the two polymorphs are summarized in Table 1.

## 3 Materials and Methods

### 3.1 Materials

Ritonavir form II was supplied by AbbVie Inc. and was used without further purification. Ethyl acetate (HPLC >99.95% purity, Fisher), toluene (reagent grade >99.7%, Sigma-Aldrich), acetonitrile (HPLC ≥99.9%, Honeywell), acetone (HPLC ≥99%, VWR International), and *n*-heptane (HPLC ≥99%, Honeywell) were used as supplied.

The metastable form I of ritonavir was prepared by the reverse addition technique in ethyl acetate/heptane (1:2 by volume) mixture^32^ as follows: (i) dissolving 10g of ritonavir form II in 40 mL of ethyl acetate at 70 °C and refluxing for at least 1 h; (ii) filtering the solution to produce a concentrated solution of ritonavir (reactant A) by using a preheated syringe equipped with 0.45 μm filter membrane; (iii) charging 80 mL of heptane with 50 mg of ritonavir form I seeds and stirring at room temperature to obtain reactant B; (iv) slowly adding reactant A to reactant B whilst constantly stirring. Since form I is the kinetically favored form and largely insoluble in heptane, it can be expected that it will always be crystallized out first in an ethyl acetate/heptane mixture as long as the solution is free of any form II contamination. In this overall procedure, maintenance of the crystallization temperature was not found to be critical.

### 3.2 Determination of Solubility as a Function of Solvent Type

The solubility of ritonavir form I in pure acetone, ethyl acetate, acetonitrile, and toluene was determined by dissolving excess amounts of ritonavir form I in 10 mL of solvent, agitated for 2 h using a MaxQ 2000 Barnstead/Lab-line shaker and maintained at a constant temperature of 10 °C using a Julabo F25 recirculating bath. The 2 h time window was selected based on the experimental observation that form I had a potential transformation risk to form II if the stirring continued for more than 3 h and also that the very low solubility of form I in these four solvents would not present a notable difference of the concentration values between 2 or 3 h of stirring. Subsequently, the supernatant was filtered through a 0.45 μm membrane filter, and the undissolved crystals were isolated. These were confirmed to be of form I by powder X-ray diffraction (PXRD), confirming that no solvent-mediated phase transformation had taken place during the time frame that encompassed the solubility measurement experiments. The extracted filtrates were weighted and then dried at 50 °C with each experiment being repeated at least three times and with the average data being used for the determination of form I solubility gravimetrically.

### 3.3 Polymorphic Screening as a Function of Solvent Type

The crystallization behavior of ritonavir form I in acetone, ethyl acetate, acetonitrile, isopropanol, and toluene solvents was investigated isothermally utilizing a Technobis Crystal 16 unit^91^ using the crash cooling approach previously described.^45,92^ Different supersaturated solutions (related to the equilibrium solubilities of form I) were prepared by dissolving the corresponding amount of ritonavir form II in 15 g of solvent. The solutions were then stirred and heated to make sure that all the crystals were dissolved completely. About 1 mL of the supersaturated solutions was then withdrawn and filtered through a preheated 0.45 membrane filter before being transferred into sample vials. These filtered solutions were then heated to 50 °C and held for 90 min before cooling to the desired crystallization temperature (10 °C) at a rate of 20 °C min^–1^. Once the solution had nucleated, the resultant crystals were filtered and oven-dried at 50 °C with each experiment being repeated at least 4 times. Note that the gentle drying process after washing removed the solvent residual from the crystal surfaces, producing dry crystals for further characterization. The polymorphic forms of the recrystallized material were identified using PXRD (Bruker D8 advanced X-ray diffractometer).

### 3.4 Measurement of Induction Times as a Function of Solution Supersaturation

The induction times to the nucleation onset point for ritonavir form I in supersaturated agitated solutions as a function of solvent were measured isothermally with the Technobis Crystal16 unit.^91^. In this, solutions of ritonavir in each of the solvents were prepared by dissolving an appropriate amount of form II consistent with the desired supersaturation in 15 g of solvent. After dissolution, the solutions were stirred for 2 h, filtered through a 0.45 μm filter membrane, and transferred to the Crystal16 vials using preheated pipettes. Each solution sample was heated to 50 °C (45 °C for acetone) at a constant (700 rpm) magnetic stirring (agitation) rate for 25 min before cooling down to the desired temperature of 10 °C at a cooling rate of 5 °C min^–1^ where it was held for 20 h. The induction times were measured on the basis of the time difference between when the selected isothermal point of 10 °C was achieved and that for the nucleation onset point. Each experiment was repeated eight times to give good induction time statistics, and the experiments were repeated over a range of supersaturations for each of the four different solvents. The crystals produced were isolated and characterized to cross check the polymorphic form by PXRD (Bruker D8 Advanced), differential scanning calorimetry (DSC) (Mettler Toledo DSC 1 STAR System), and scanning electron microscopy (SEM) (Carl Zeiss EVO MA15).

### 3.5 Induction Time Analysis Using Classical Nucleation Theory

Measured induction times were analyzed using CNT to calculate the nucleation rate (*J*) through the Arrhenius relationship as follows
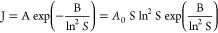
1where *A* is the pre-exponential kinetic factor, *A*_0_ is the pre-exponential kinetic factor constant, *S* is the supersaturation given by
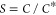
2and the thermodynamic parameter, *B*, is given by
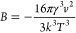
3where *C* is the solute concentration, C* is the equilibrium solubility, *k* is the Boltzmann constant, *T* is the nucleation temperature, γ is the effective interfacial energy, *v* is the molecular volume.

*A*_0_ and *B* were derived from the intercept and slope, respectively, of a linear fitting of  versus .

The attachment rate, *f* × *C*_0_, was estimated by
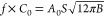
4

From the CNT, the critical size, *r*_c_, for the nucleation cluster was calculated from
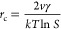
5

Note that further details can be found in S1, Supporting Information.

### 3.6 MD Simulations and Free Energy Calculations

**Figure 3:**
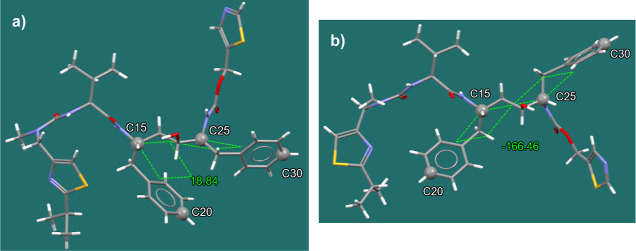
Definition of the dihedral torsion (C20–C15–C25–C30) angles of the phenyl conformations with their angles of 188.84° and −166.46° for the ritonavir form I (a) and form II (b) molecules.

The conformational landscapes of ritonavir were assessed to reflect the tendency of large drug-like molecules to exhibit solvent-dependent conformational states.^25^ To study conformational ensembles of ritonavir, enhanced MD simulations with explicit solvent to capture the unique solvent–solute interactions were carried out for the four solvent systems (acetone, ethyl acetate, acetonitrile, and toluene) in which form I crystallized, and for ethanol, where form II crystallized.^26^ From MD simulation results, the dihedral torsion (C20–C15–C25–C30) angles of the phenyl substituents, as defined in Figure 3, were analyzed using a histogram to characterize the conformational states of ritonavir, which included the cis, trans, and an intermediate conformation to probe for any changes to the intermolecular chemistry associated with solvation. From the MD simulations, the dihedral torsion (C20–C15–C25–C30) angles of the phenyl groups (defined as phenyl conformation) that exhibited a dihedral torsion angle between −60 and 60° were classified as the cis phenyl conformation. The form I molecular structure (Figure 3a) has a trans carbamate conformation with a phenyl torsion angle of 18.84° (cis phenyl conformation), and it may be expected to have a high probability of correlating cis phenyl and trans carbamate conformations. Those angles lying in the range from −120 to −60° or 60 to 120° were classified as intermediate, and those in the range from −180 to −120° or 120 to 180° were classified as trans phenyl conformation. As shown in Figure 3b, the molecular structure of form II has a phenyl conformation angle of −166.46° (trans phenyl conformation) with a cis carbamate conformation, which may suggest a possible link between cis carbamate conformation and trans phenyl conformation.

These MD simulations were performed using the replica exchange solute tempering approach^93^ with the OPLS-4 force field,^94^ using the Desmond MD engine,^95^ as implemented in the Schrodinger 2023–3 software suite.^96^ MD simulations were run for 100 ns, with 8 evenly spaced temperature replicas ranging from 300 to 665 K. Additionally, solvation free energies for all the solvents were computed using free energy perturbation,^97^ with 20 ns of production simulation time with 18 lambda windows.

## 4 Results and Discussion

### 4.1 Crystallization Outcomes

**Figure 4:**
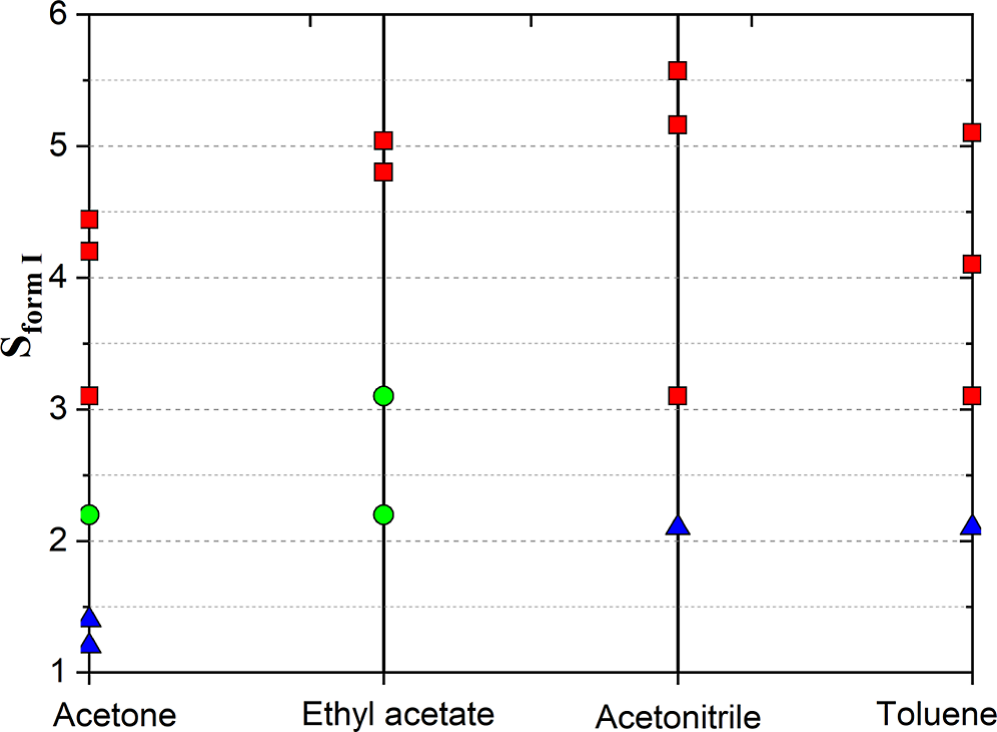
Crystallization outcomes as a function of supersaturation and solvent at 283.15 K (red square—form I; green circle—form II; blue triangle—no crystallization after two days).

Figure 4 shows the results of the crash cooling experiments carried out isothermally at 283.15 K following crystallization from polar aprotic (acetone, ethyl acetate, and acetonitrile) and apolar (toluene) solvents as a function of supersaturation. The measured solvent and saturation-dependent induction times were found to be very long, varying from hours to days. Note that the very long induction times observed in this study with different solvents and supersaturations demonstrate the low nucleation rate and crystallizability. The resultant polymorphic forms of the crystals were found to be independent of the choice of solvent, with the metastable form I preferred at the higher supersaturations and the stable form II favored at the lower supersaturations. This behavior is consistent with Ostwald’s Rule^98^ and with other studies, e.g., l-glutamic acid^15,50^ regarding the typical behavior for a polymorphic system.

Crystallization from polar protic (ethanol) solvents was not found to be successful due to both their high solubility and solution viscosity, as these tend to be characterised by low crystallisability (low nucleation rate) for those systems and concomitantly unrealistically very long induction times. Hence, it was not found to be feasible to carry out detailed studies on this system.

### 4.2 Identification of the Polymorphic Form

**Figure 5:**
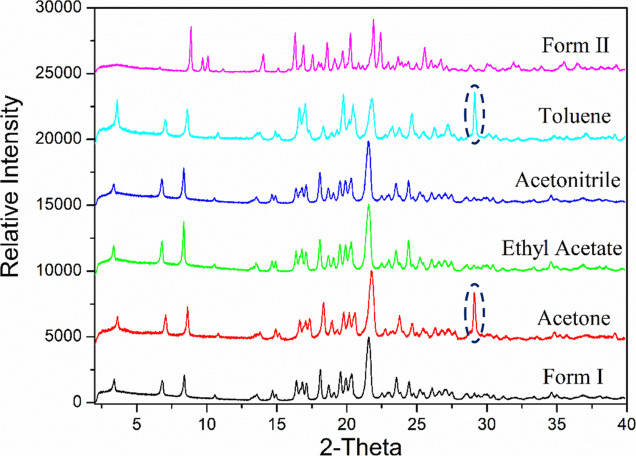
PXRD patterns of ritonavir nucleation in different solvents, confirming the recrystallization of form I from these solvents. Note that the extra peak cycled was found to be from the sample holder.

Figure 5 shows the PXRD patterns for the recrystallized materials recovered from the various solvents, revealing that the nucleated solids prepared from acetone, ethyl acetate, acetonitrile, and toluene solutions have the same form as the reference material prepared by recrystallization from the ethyl acetate–heptane mixture. In this, the characteristic diffraction peaks of form I located at 3.30, 6.80, 8.40, and 24.50 2θ can be easily distinguished from those of form II (characteristic peaks at 9.51, 9.88, and 22.20 2θ).^44,84,85^ Note that the existence of the extra peak at ca. 28° 2θ is due to the sample holder and not the analyte. To the best of our knowledge, this is the first time that the so-called “disappearing form I polymorph” has been isolated using the crash cooling approach from form II source materials. This may reflect the higher solubility and available solution supersaturation of the metastable form I in organic solvents and would be consistent with the metastable form I of ritonavir being the kinetically favored form. In this, at the nucleation on-set point, the higher supersaturations would produce smaller critical cluster sizes, stabilizing the metastable form.^50,72,99^ The PXRD analysis is supported by reference DSC data for forms I and II of ritonavir which are given in Figure S1 (Supporting Information) and reveal endothermic melting peaks at around 123 °C for form I and 126 °C for form II.^84,100^ The observed melting points for the form I isolated from the four solvents were found to fluctuate slightly from 123 °C in toluene to 125 °C in acetone with their enthalpies of fusion calculated based on the DSC data increasing from 38.43 to 41.19 kJ mol^–1^ and the full widths at half-maximum decreasing from 3.45 to 2.21 °C [see Table S1 (Supporting Information) for further details], reflecting, perhaps, the different crystallinity and morphology of the associated solid forms.

**Figure 6:**
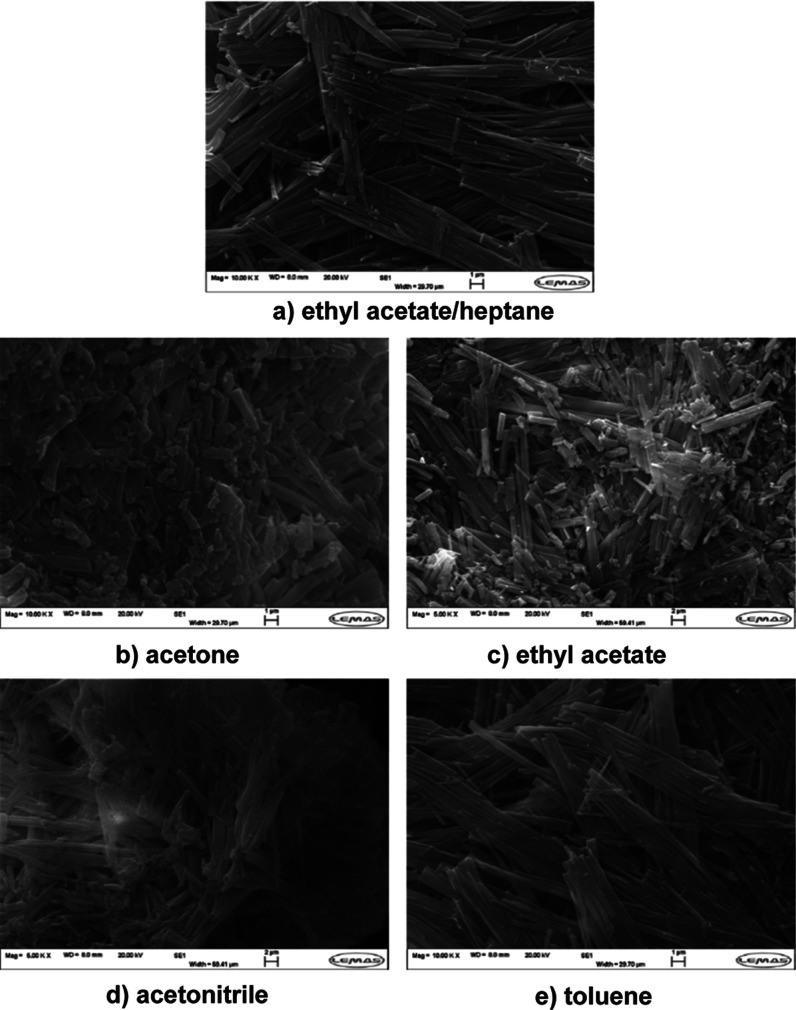
SEM micrographs of ritonavir crystals produced following nucleation in different solvents: (a) form I reference; (b) acetone; (c) ethyl acetate; (d) acetonitrile, and (e) toluene. Note that the top SEM image shows the reference for form I, i.e., as crystallized from ethyl acetate/heptane mixture with a volume ratio of 1:2, which has a quite different morphology from other four solvents.

The morphological data for the recrystallized materials is shown in Figure 6 with the SEM data revealing an elongated plate-like morphology for the form I crystals isolated from acetone and acetonitrile solutions while the crystals were found to be more needle-like in ethyl acetate, ethyl acetate/heptane and toluene. Previous studies^28^ have found that polar solvents such as acetone produced a more elongated plate-like morphology of ritonavir form I while apolar toluene has more needle-like crystals, hence a higher aspect ratio. This is consistent with the formation of strong π–π stacking interaction between the aromatic rings on the side faces of ritonavir crystal and the phenyl ring in the toluene.^28^ Overall, the form I crystals tended to be very small (ca. 10 × 0.5 μm), while the form II crystals were found to be larger and more elongated (ca. 600 × 15 μm). The solvent selection was observed to have a significant effect on the crystal morphology of form II crystals, where apolar solvents were found to significantly increase the aspect ratio. Despite this, forms I and II cannot be distinguished solely on their observed crystal morphology since the crystals of form II may also exhibit a needle-like crystal habit.^28^ Since form I was found to be the only solid form crystallized in the above four solvents, the analysis of the influence of solvent on the nucleation kinetics were focused on form I in this study.

### 4.3 Determination of Solubility and Nucleation Propensity

**Figure 7:**
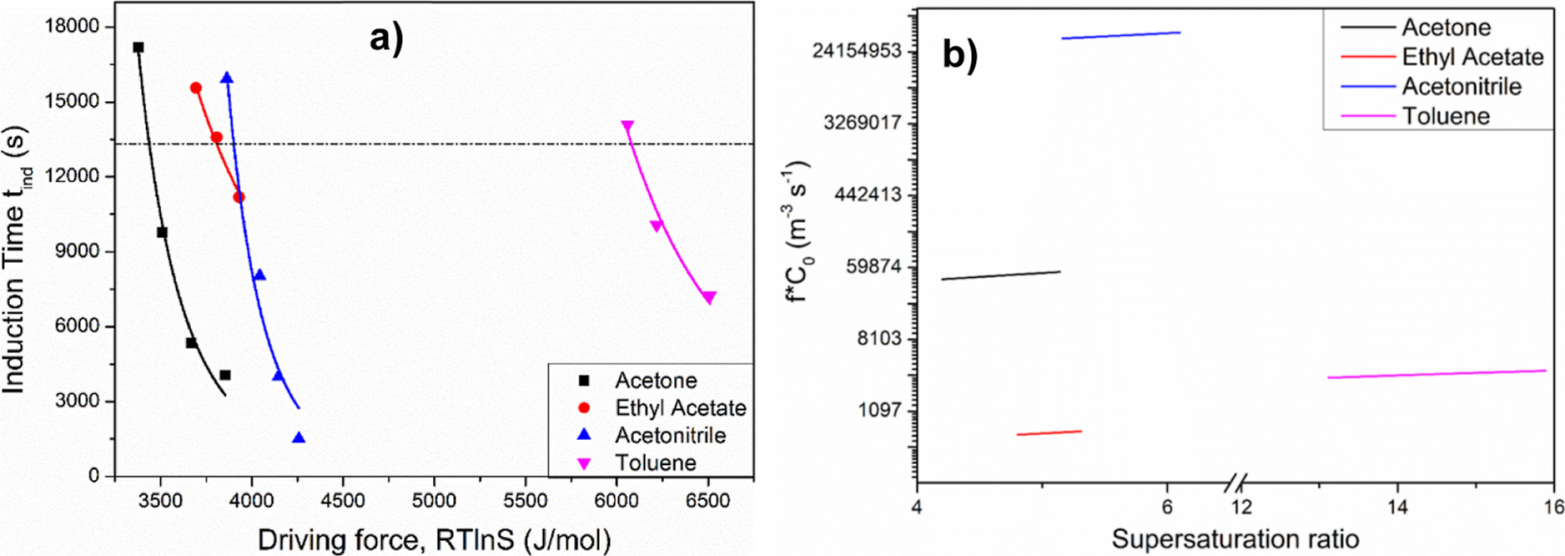
(a) Induction time versus nucleation driving force in different solvents. Lines represent exponential fits. Dashed line represents the equal induction time at *t* = 13 320 s to quantitatively highlight the influence of solvent on the nucleation process; (b) dependence of the molecular attachment rate on the supersaturation ratio of ritonavir form I in four solvents.

Table 2 summarizes the solubility and nucleation propensity of ritonavir form I at 283.15 K and the solvation free energy produced from MD FEP calculations, revealing that the solubility of form I increases in the order of toluene, acetonitrile, ethyl acetate, and acetone. As the nucleation rate highly depends on the supersaturation and the supersaturation ranges for the four solvents did not really overlap, it was not feasible to directly compare their nucleation rates. However, at a supersaturation of around 5, the nucleation rates in acetone, ethyl acetate, acetonitrile, and toluene were found to decrease with the values of 245.79, 73.61, 62.79, and 11.51 m^–3^ s^–1^, respectively, as shown in Table 2 (column 6) and Section S3 (Supporting Information). The value for toluene was estimated by fitting the data with an exponential function [Figure S2 (Supporting Information)]. Figure 7a shows the associated induction times (*t*_ind_) to the nucleation on-set point as plotted against the nucleation driving force *RT* ln *S* for each of the four solvents. The data revealed a direct correlation between the measured solubilities and an increasing difficulty of nucleation, reflecting a higher driving force, as a function of solvent selection in the order of acetone, ethyl acetate, acetonitrile, and toluene.

**Table 2:** Nucleation Parameters for Ritonavir Form I in Different Solvents at 283.15 K According to the Classical Nucleation Theory and Solvation-Free Energy from MD Simulations

solvent system	solubility × 10^3^ (mole fraction)	saturated concentration × 10^3^ (g g^–1^)	*S*	*RT* ln *S* (J mol^–1^)	nucleation rate (m^–3^ s^–1^)	critical nucleus radius (Å)	number of molecules	ln *A*_0_ [ln (m^–3^ s^–1^)]	solvation free energy (kcal mol^–1^)
ethanol	N/A	N/A	N/A	N/A	N/A	N/A	N/A	N/A	–38.89
acetone	4.30	224.42	4.20	3378.35	58.17	10.93	6.06	6.33	–37.48
		236.99	4.44	3509.16	102.38	10.53	5.41		
		253.54	4.75	3668.04	187.15	10.07	4.73		
		274.35	5.14	3853.80	245.79	9.59	4.08		
ethyl acetate	1.33	52.24	4.80	3692.69	64.27	6.26	1.13	2.58	–36.78
		54.85	5.04	3807.55	73.61	6.07	1.04		
		57.79	5.31	3930.40	89.43	5.88	0.94		
acetonitrile	0.66	59.81	5.16	3862.94	62.79	14.20	13.27	12.22	–33.98
		64.56	5.57	4042.94	250.29	13.57	11.57		
		67.46	5.82	4146.29	124.45	13.23	10.73		
		70.71	6.10	4256.91	659.70	12.88	9.91		
toluene	0.22	19.40	13.11	6058.01	70.95	7.32	1.82	2.18	
		20.76	14.03	6217.67	99.32	7.14	1.68		–33.08
		23.52	15.89	6510.74	138.07	6.81	1.47		

Estimation of the attachment rate (*f* × *C*_0_) of building units to a nucleus is plotted in Figure 7b together with its variation of *f* × *C*_0_ with supersaturation and solvent. In literature, the value of attachment rate can vary from 10 to 10^11^ m^–3^ s^–1^ according to the estimation by Sullivan et al.^47^ based on the nucleation kinetic data of small molecule compounds (benzoic acid in toluene and PABA in 2-propanol, ethyl acetate, and acetonitrile). As shown in Figure 7b, the value for ritonavir solutions varying from 10^2^ to 10^7^ m^–3^ s^–1^ determined in this study was found to align well with the literature; hence, this was considered appropriate to be used for the evaluation of the molecular kinetic process of ritonavir solutions in the four solvents. It was found that the attachment rates of ritonavir molecules in acetonitrile solutions were the highest being observed to be about 3 orders of magnitude higher when compared with acetone (the second highest), followed by toluene and then ethyl acetate. These data are consistent with the nucleation rate, critical nucleus radius, and number of molecules in a critical nucleus summarized in Table 2 (columns 6, 7, and 8).

Table 3 provides a direct comparison between the solvent-dependent data in order to quantify the inter-relationship between solution properties and the crystallizability of the materials. Analysis of the supersaturation dependence of the nucleation rates reveals that in order to reach an equal induction time of *t*_ind_ = 13320*s*, the driving forces for nucleation were 3437 J mol^–1^ in acetone, 3806 J mol^–1^ in ethyl acetate, 3900 J mol^–1^ in acetonitrile, and 6079 J mol^–1^ in toluene, consistent with the reverse order of their solubility and also nucleation rates (Table 2) as well as the crystallizability of ritonavir form I.

**Table 3:** Nucleation Parameters and Some of the Solvent Properties

	driving force (J mol^–1^)	γ (mJ m^–2^)	boiling point (°C)	viscosity (mPa s)	dipole moment (*D*)	dielectric constant
acetone	3437	3.39	56.53	0.33	3.00	20.60
ethyl acetate	3806	2.12	77.00	0.46	1.60	6.02
acetonitrile	3900	5.04	81.60	0.38	3.92	37.50
toluene	6079	4.08	110.63	0.59	0.36	2.38

Calculation of the solvation energies from MD simulations, as shown in Table 2 (column 10), reveals a direct correlation between the solvation energy and, by implication, the desolvation energy barrier associated with nucleation and solute solubility for the different solvents. The slope of solvation energy correlation with solubility was found to be about 1.5. While these results are quite consistent with previous studies on other systems, it should be pointed out that these encompass studies on compounds with much lower molecular weights and also different solvents.^11,45,61,66^ Hence, this work provides, perhaps, the first observation of such a trend in relative solubilities for a high molecular mass representative pharmaceutical material, such as ritonavir.

### 4.4 Influence of Solvent Properties on Nucleation

**Figure 8:**
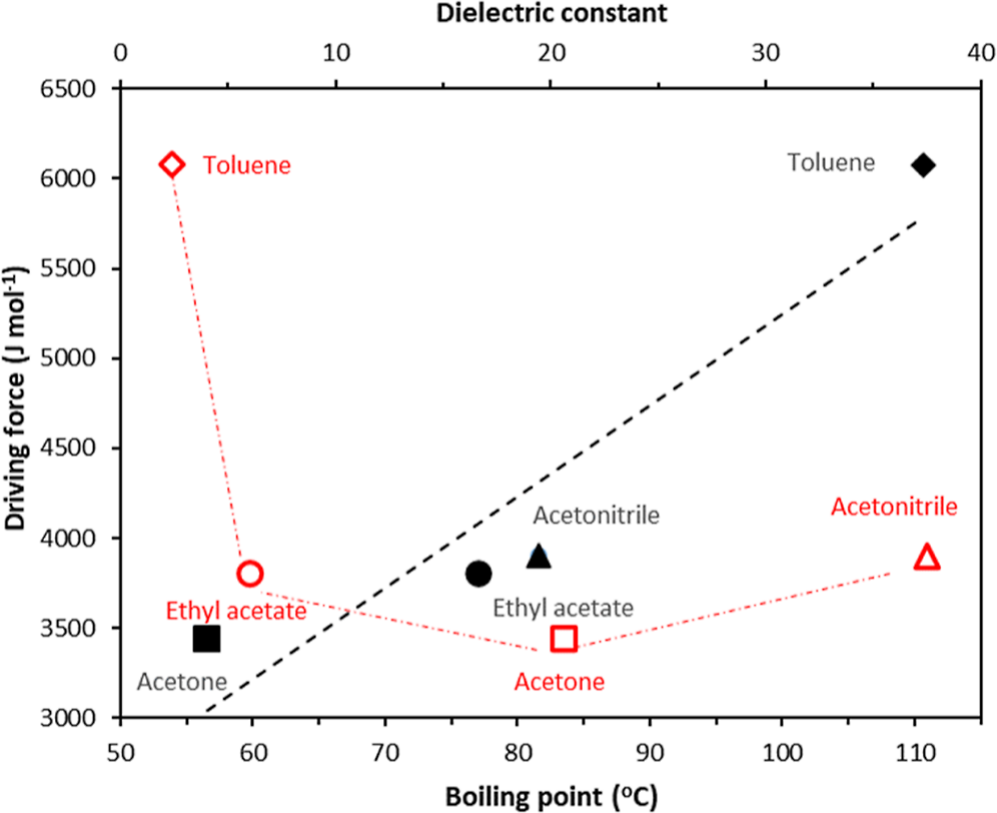
Driving force vs solvent boiling point and dielectric constant (red) for four solvents. The black straight line and red lines are guides for the eye.

Examination of the data, summarized in Tables 2 and 3, reveals ritonavir crystallization from acetonitrile solutions to have the largest pre-exponential kinetic parameter, followed by acetone, ethyl acetate, and toluene. This behavior correlates well with a number of other physical chemical parameters such as the solvent energy/desolvation calculations from MD as well as solvent polarity (Table 3) with the pre-exponential factor decreasing with the decreasing value of dipole moment and dielectric constant. The correlation between driving forces to nucleation onsets versus solvent boiling points (black colored symbols) is plotted in Figure 8. These reveal that generally, the solvent boiling points can be taken as an effective measure of solvent/solvent intermolecular forces and hence proportional to the enthalpy of vaporisation^60^ and overall were found to be consistent with the nucleation propensity being strongly dependent on the strengths of solute/solvent interactions. The driving force as a function of solvent dielectric constant (red colored symbols in Figure 8) shows that apolar toluene solutions require much higher driving force for ritonavir molecules to be nucleated with respect to other polar aprotic (acetone, ethyl acetate, acetonitrile) solvents, consistent with the lowest solubility and nucleation rate in toluene solutions, which may be due to the ring stacking interactions between ritonavir and toluene molecules.

### 4.5 Molecular Dynamics Simulations for Examining Solute–Solvent Interactions

**Figure 9:**
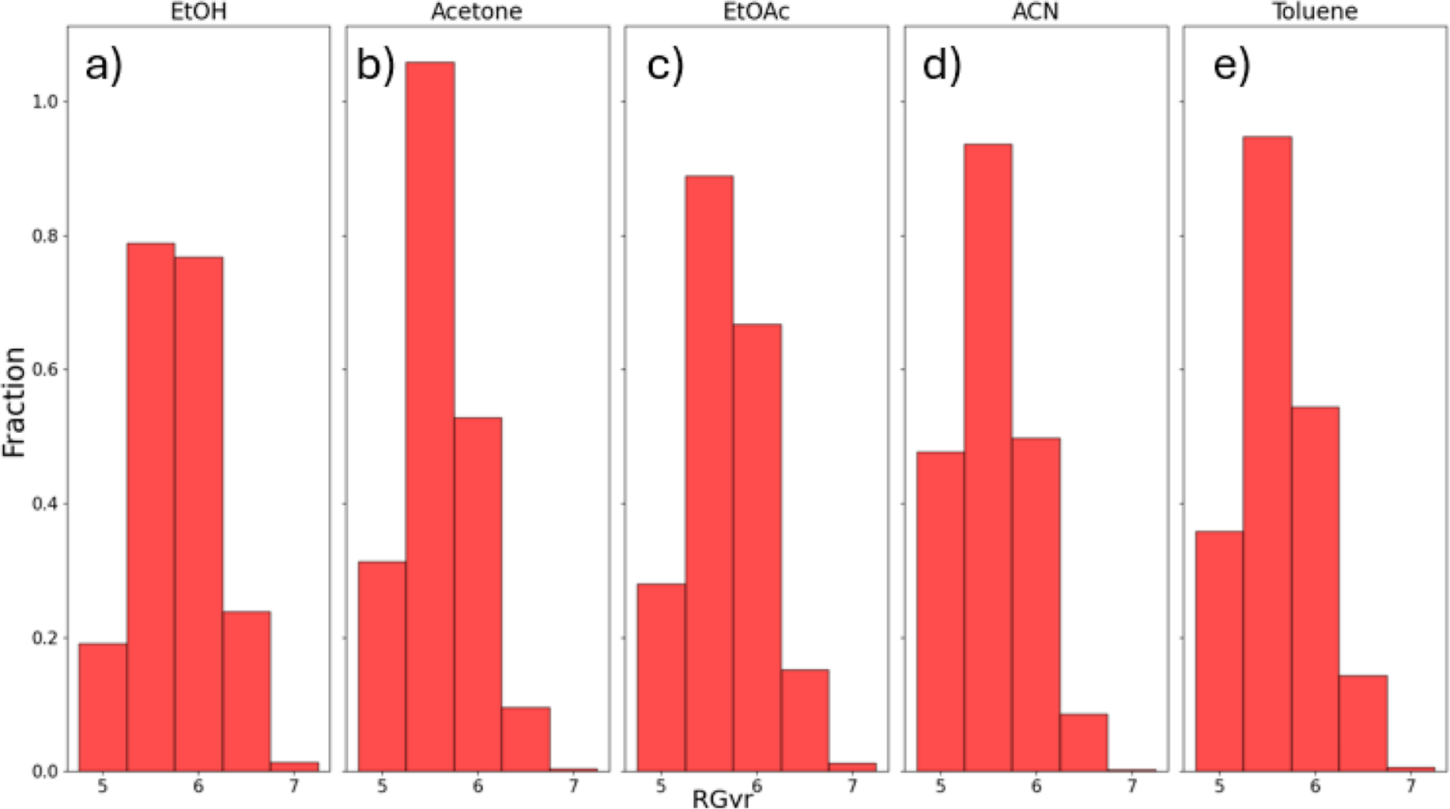
(a–e) Distributions of radius of gyration observed for each solvent system showing a range of dynamic extended and folded conformations.

MD simulations, using explicit solvent, were conducted to understand the conformational preferences of ritonavir in the different crystallization solvents. Through MD simulations, a wide range of dynamic conformations were observed in all solvent systems, including both extended and folded conformations, as observed by the distributions of their radius of gyration (R-Gyr) (Figure 9), which quantifies the degree of molecular folding. Similarly, in NMR studies conducted by Augustyniak et al.,^101^ various extended and folded conformations were observed for ritonavir in EtOH. Interestingly, in EtOH where Form II favorably crystallizes, relative higher populations of extended conformations, as shown by the larger R-Gyr populations. When comparing the conformations generated from the MD trajectories to the specific crystal conformations in Form I and Form II, noticeable root-mean-square deviations (Table 4) were observed for each system. As suggested by Augustyniak et al.,^101^ the specific crystal conformation for either polymorph and so the exact alignment of torsion angles is not easily observed in solution which might suggest the likelihood of global conformational strain reflecting dynamic nature of the molecular and intramolecular interactions in solution.

**Table 4:** Average RMSD (in Å) between the Form I and Form II Conformations and the Observed Conformations in the MD Trajectory

solvent	form I	form II
EtOH	7.38	7.41
acetone	7.24	7.34
ethyl acetate	7.28	7.31
acetonitrile	7.35	7.29
toluene	7.32	7.36

**Figure 10:**
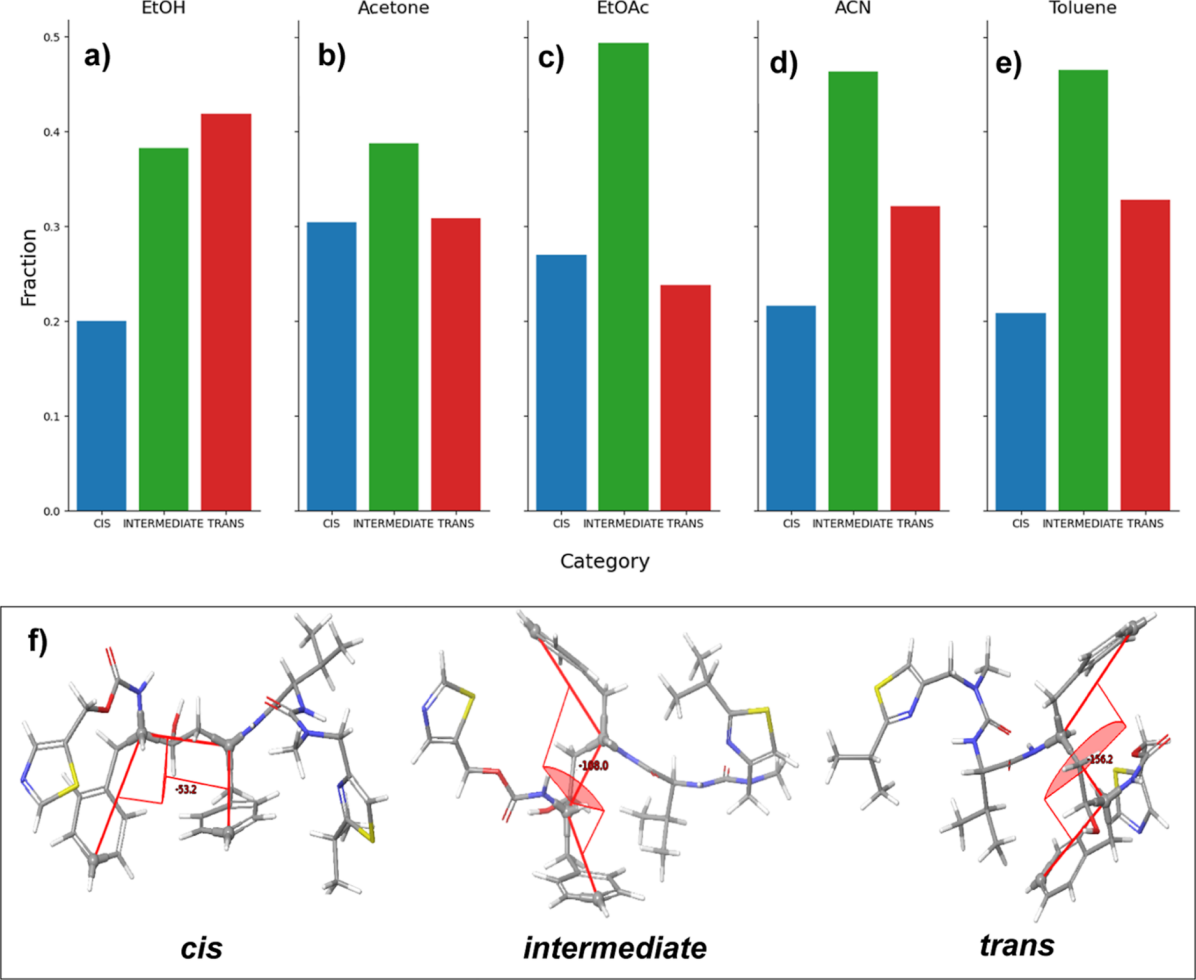
(a–e) Histogram of the observed ritonavir phenyl conformations throughout the MD simulations for each solvent. (f) Example depictions of conformers in the cis, intermediate, and trans conformations.

While many structurally diverse conformations were observed in the MD trajectories, the specific local structural features observed in different solvents may provide insights into solution conformational preferences, which may lead to different polymorphic outcomes. To bridge findings from the MD conformational ensembles in various solvents and the observed crystal structure conformations, distinct conformational features obtained from each solvent were assessed as they related to the crystal conformation. The significant differentiating features from the MD simulation analysis include the observation of the intramolecular hydrogen bond (IMHB) between the hydroxyl (donor) and carbamate (acceptor) groups, as relative phenyl conformations within the molecule (Figure 10a). These features and atoms can be hypothesized to play an important role in the nucleation of the two polymorphs, as they play a key roles in the different intermolecular interaction patterns observed between form I and form II, as shown in Figure 2.

**Figure 11:**
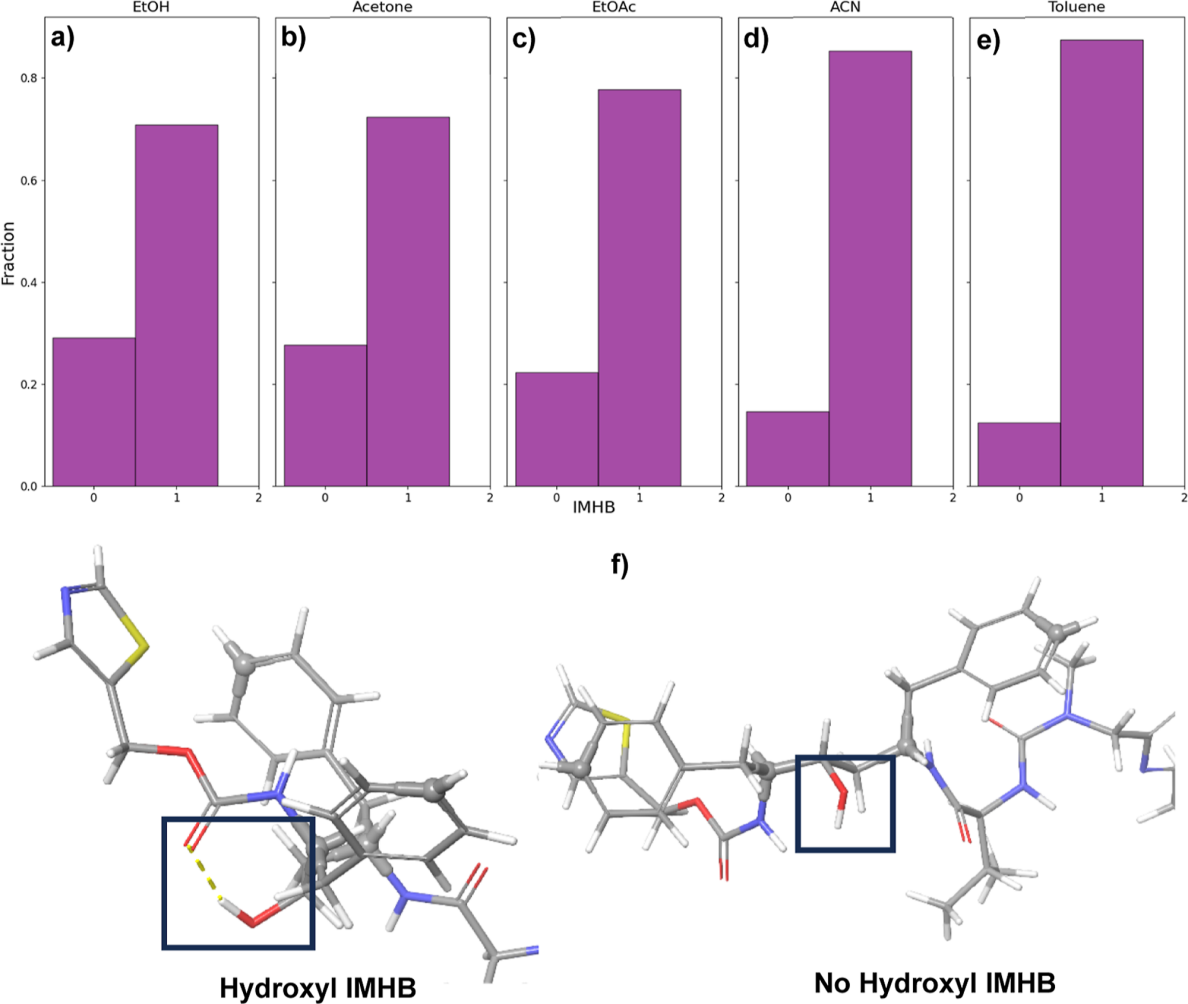
(a–e) Histogram of intramolecular hydrogen bonding observed (IMHB = 1) and not observed (IMHB = 0) for the hydroxyl group throughout the MD simulations; (f) structural depiction of (left) the hydroxyl group forming an IMHB with the carbamate oxygen and (right) the free hydroxyl group without IMHB.

In the MD simulations with ethanol, similar to the conformer in form II, a dominant trans configuration of the phenyl conformation was observed (Figure 10a). This was accompanied by a significant population (30%) of phenyl conformations lacking IMHB (Figure 11a). These findings may explain the favorable crystallization of form II from this solvent system, as previously described by Bauer et al.^26^ Here, the preferential trans conformation may also facilitate the aromatic stacking patterns observed in form II, which has been hypothesized to play a key role in nucleation.^102^ In addition, the relatively lower populations and weaker interactions of the hydroxyl-carbamate IMHB may better allow the intricate form II hydrogen bonding networks to form, as these interactions need to be broken.

The MD simulations with acetone (Figure 10b) and EtOAc (Figure 10c) also reveal a notable population of conformers that still lacked the IMHB (Figure 11b,c), and an intermediate configuration between trans and cis phenyl conformations became predominant. From this, it would seem to be likely that crystallization of ritonavir from acetone and EtOAc may result in either form I (trans phenyl with less intricate intermolecular H-bonding) or form II (cis phenyl with more intricate intermolecular H-bonding), i.e., as consistent with experimental observations from the crystallization experiments (Figure 4).

While the MD simulations involving acetonitrile (Figure 10d) and toluene (Figure 10e) also revealed a dominant intermediate configuration, most of the observed intermediate phenyl conformations also exhibited IMHBs (Figure 11d,e). This preference for IMHB in these two solvents may explain the preferred crystallization of form I, in which the formation of form II relies on an intricate intermolecular hydrogen bonding network involving the hydroxyl group. Examinations of the HB networks (Figure 2) in the crystal structures of both forms found that for form I, the three homo intermolecular HBs lead to a ″dimerized” intermolecular stacking structure (Figure 2a), while for form II, the four hetero and much more conjugated intermolecular HBs formed a ring-like structure (Figure 2b), hence more interdependent on each other. Therefore, over 90% of phenyl conformations in these two solvents were found to have IMHBs, indicating significant disruption of forming a ring-like intermolecular HB network (Table 1) in form II, leading to the preference of form I. Moreover, the strong IMHB of the hydroxyl hydrogen would also be expected to shield the carbamate oxygen in these solvents which may, in turn, potentially hinder the nucleation of form II. Here, the nucleation of Form II would simultaneously require not only the breaking of the strong IMHB and the three homo HBs involved in form I’s multiple dimeric stacking interactions but also require the assembly of form II’s intricate four-membered ring-like intermolecular hetero HB network. Such a process could be expected to be kinetically limited as the process of breaking the strong IMHB interactions in these solvents and the formation of an intricate HB network has to occur simultaneously.

Overall, the MD simulation results are consistent with the experimental findings as shown in Figure 4, i.e., both forms I and II of ritonavir may be crystallized from acetone and ethyl acetate, while acetonitrile and toluene preferably tend to crystallize ritonavir form I. Overall, such inhibition may be due to the hindrance of form II by the IMHB formation being stronger, given the more complete heterohydrogen bonding network in form II.

### 4.6 Discussion

The preparation of form I isothermally from the crash cooling method in different solvents is in good agreement with those in previous studies. Chemburkar et al.^32^ employed a reverse addition technique, which can result in a very high driving force for nucleation, to make the “disappeared polymorph” reappear. According to the structure and solubility analysis, Bauer et al.^26^ pointed out that the primary nucleation of ritonavir from solution should follow Ostwald’s rule^98^ and that the metastable form I should crystallize preferentially. However, these previous studies did not provide any quantitative characterization of the crystallizability of the material, notably through an analysis of neither the nucleation kinetics, the MSZW for form I, nor any correlations between nucleation propensity and the growth solution’s molecular-scale interactions. Through the combination of the nucleation kinetic and the molecular simulation data reported here, it is possible to fully characterize more completely the links between solvent properties, solvation, inter/intramolecular interactions in solution, and the nucleation process.

The nucleation kinetics experiments suggest that for a given induction time, the driving force required for nucleation in different solvents increases following the order of acetone, ethyl acetate, acetonitrile, and toluene. This work further showed that the interfacial energies which were calculated from CNT did not have any direct correlation to the nucleation propensity. The variation in the interfacial energy for ritonavir in the different solvents was found to be relatively high (Table 3), ranging from 2.12 mJ m^–2^ in ethyl acetate to 5.04 mJ m^–2^ in acetonitrile.

The nucleation rate, critical nuclei size, number of nuclei, and effective surface energy obtained from this study were found to be generally comparable with these compounds from literature although these tend to be much smaller molecular compounds when compared to ritonavir, as shown in (Table S2, Supporting Information), with two compounds (methyl stearate and tolfenamic acid form II) having much higher nucleation rates obtained^103,104^ with progressive nucleation mechanism, and the supersaturation values used for nucleation studies in the current study being several times higher than other compounds from literature.

## 5 Conclusions

The solubility of form I in the four solvents was found to increase on the order of toluene, acetonitrile, ethyl acetate, and acetone. For the same induction time, the nucleation driving force was found to decrease in the same order, i.e., in reasonable agreement with the decrease in solubility and the increase in boiling point for these solvents. The attachment rates of ritonavir molecules in the four solvents were found to decrease in the order acetonitrile > acetone > toluene > ethyl acetate, consistent with the critical nucleus radius, number of molecules in a critical nucleus, and nucleation rate. However, no clear correlations between solvent selection and the interfacial energies were found.

The MD simulation studies demonstrated that the populations of intramolecular and intermolecular hydrogen bonding involving the hydroxyl hydrogen in different solvents may play a role, among other factors such as solvent composition and supersaturation, in the control of the formation of ritonavir forms I and II. The dominant “trans” conformations of the phenyl orientations, found in ethanolic solutions, may also suggest a preference to crystallize form II, while the intermediate configurations of molecular conformations with dominant intramolecular hydrogen bonding in acetonitrile and toluene would be expected to lead to the formation of form I. The roughly balanced populations of intermolecular and intramolecular hydrogen bonding formed in acetone and ethyl acetate may explain the crystallization of either form I or form II. These detailed analyses of the solvation and desolvation among the molecules of ritonavir within a range of different (apolar, protic polar, and aprotic polar) solution environments provided a significantly improved understanding at the molecular scale as to how the solution chemistry, solute/solvent molecular properties, and crystallization environment influence the crystallizability, nucleation kinetics, and polymorphic outcomes of this highly representative pharmaceutical compound within the overall crystallization and particle design process.

## Supporting Information Available

Further details on induction time analysis using classical nucleation theory; DSC patterns of ritonavir nucleation in four solvents; fitting of nucleation rates in toluene with supersaturation for estimating the nucleation rate at *S* = 5; melting points, enthalpies of fusion, and fwhm for four solvents; and comparisons of relevant nucleation properties of ritonavir form I in different solvents and other compounds (PDF)

## List of Symbols and Abbreviations

*A*: pre-exponential kinetic factor (m^–3^ s^–1^)
*A*_0_: pre-exponential kinetic factor constant (m^–3^ s^–1^)
*B*: thermodynamic parameter (K^3^)
*C*: concentration in solution (g g^–1^ solvent)
*C**: solubility at the crystallization temperature (g g^–1^ solvent)
*C*_0_: concentration of nucleation sites
*f**: attachment frequency
*f* × *C*_0_: attachment rate
*J*: nucleation rate (m^–3^ s^–1^)
*r*_c_: critical nucleus size (Å)
*RT* ln *S*: nucleation driving force
*S*: supersaturation
*t*_ind_: induction time of nucleation (s)
*T*: nucleation absolute temperature (K)
*v*: molecule volume (m^3^)
γ: effective interfacial energy (mJ m^–2^)
*k*: Boltzmann constant (J K^–1^)
ACN: acetonitrile
AMBA: 4-amino-3-methoxybenzoic acid
ANBA: 4-amino-3-nitrobenzoic acid
API: active pharmaceutical ingredient
CNT: classical nucleation theory
DSC: differential scanning calorimetry
EtOAc: ethyl acetate
EtOH: ethanol
FTIR: Fourier-transform infrared spectroscopy
HB: hydrogen bond
IMHB: intra-molecular hydrogen bonding
KBHR: Kashchiev–Borissova–Hammond–Roberts
MD: molecular dynamics
PABA: *para*-amino benzoic acid
PXRD: powder X-ray diffraction
QA: quality assurance
SEM: scanning electron microscope

## References

[ref1] Du W.; Yin Q.; Hao H.; Bao Y.; Zhang X.; Huang J.; Li X.; Xie C.; Gong J. Solution-mediated polymorphic transformation of prasugrel hydrochloride from form II to form I. Ind. Eng. Chem. Res. 2014, 53 (14), 5652–5659. 10.1021/ie404245s.

[ref2] Maher A.; Croker D. M.; Rasmuson Å. C.; Hodnett B. K. Solution mediated polymorphic transformation: form II to form III piracetam in ethanol. Cryst. Growth Des. 2012, 12 (12), 6151–6157. 10.1021/cg301290z.

[ref3] Bernstein J. International Union of Crystallography monographs on crystallography. In Polymorphism in Molecular Crystals; Oxford University Press, 2002; .

[ref4] Haleblian J.; McCrone W. C. Pharmaceutical Applications of Polymorphism. J. Pharm. Sci. 1969, 58 (8), 911–929. 10.1002/jps.2600580802. 4899118

[ref5] McCrone W. C. Polymorphism; Fox D., Labes M. M., Weissberger A., Eds.; Interscience Publishers: London, UK, 1965; Vol. 2, pp 725–767.Physics and Chemistry of the Organic Solid State II

[ref6] Bernstein J. Conformational polymorphism. III. The crystal and molecular structures of form II and form III of iminodiacetic acid. Acta Crystallogr. 1979, 35, 360–366. 10.1107/S056774087900354X.

[ref7] Anuar N.; Yusop S. N.; Roberts K. J. Crystallisation of Organic Materials from Solution: A Molecular, Synthonic and Crystallographic Perspective. Crystallogr. Rev. 2022, 28, 97–215. 10.1080/0889311X.2022.2123916.

[ref8] Bernstein J. International Union of Crystallography monographs on crystallography. In Polymorphism in Molecular Crystals; Oxford University Press, 2002; .

[ref9] Cruz-Cabeza A. J.; Bernstein J. Conformational polymorphism. Chem. Rev. 2014, 114 (4), 2170–2191. 10.1021/cr400249d. 24350653

[ref10] Griesser U. J.; Jetti R. K. R.; Haddow M. F.; Brehmer T.; Apperley D. C.; King A.; Harris R. K. Conformational polymorphism in oxybuprocaine hydrochloride. Cryst. Growth Des. 2008, 8 (1), 44–56. 10.1021/cg070590d.

[ref11] Liu Y.; Ma C. Y.; Gong J.; Roberts K. J. The Influence of Solvent Selection upon the Crystallizability and Nucleation Kinetics of Tolfenamic Acid Form II. Cryst. Growth Des. 2023, 23 (8), 5846–5859. 10.1021/acs.cgd.3c00450. PMC10401637 37547878

[ref12] Sacchi P.; Reutzel-Edens S. M.; Cruz-Cabeza A. J. The unexpected discovery of the ninth polymorph of tolfenamic acid. CrystEngComm 2021, 23, 3636–3647. 10.1039/D1CE00343G.

[ref13] Mattei A.; Li T. Polymorph Formation and Nucleation Mechanism of Tolfenamic Acid in Solution: An Investigation of Pre-nucleation Solute Association. Pharm. Res. 2012, 29, 460–470. 10.1007/s11095-011-0574-7. 21879384

[ref14] López-Mejías V.; Kampf J. W.; Matzger A. J. Polymer-Induced Heteronucleation of Tolfenamic Acid: Structural Investigation of a Pentamorph. J. Am. Chem. Soc. 2009, 131, 4554–4555. 10.1021/ja806289a. 19334766 PMC2729806

[ref15] Du W.; Cruz-Cabeza A. J.; Woutersen S.; Davey R. J.; Yin Q. Can the study of self-assembly in solution lead to a good model for the nucleation pathway? The case of tolfenamic acid. Chem. Sci. 2015, 6 (6), 3515–3524. 10.1039/C5SC00522A. 29511513 PMC5814770

[ref16] Tang W.; Mo H.; Zhang M.; Parkin S.; Gong J.; Wang J.; Li T. Persistent Self-Association of Solute Molecules in Solution. J. Phys. Chem. B 2017, 121 (43), 10118–10124. 10.1021/acs.jpcb.7b07763. 29017013

[ref17] Case D. H.; Srirambhatla V. K.; Guo R.; Watson R. E.; Price L. S.; Polyzois H.; Cockcroft J. K.; Florence A. J.; Tocher D. A.; Price S. L. Successful Computationally Directed Templating of Metastable Pharmaceutical Polymorphs. Cryst. Growth Des. 2018, 18, 5322–5331. 10.1021/acs.cgd.8b00765.

[ref18] Tang W.; Sima A. D.; Gong J.; Wang J.; Li T.; Li T. Kinetic Difference between Concomitant Polymorphism and Solvent-Mediated Phase Transformation: A Case of Tolfenamic Acid. Cryst. Growth Des. 2020, 20, 1779–1788. 10.1021/acs.cgd.9b01503.

[ref19] Mattei A.; Mei X.; Miller A.-F.; Li T. Two Major Pre-Nucleation Species that are Conformationally Distinct and in Equilibrium of Self-Association. Cryst. Growth Des. 2013, 13, 3303–3307. 10.1021/cg401026j.

[ref20] Mattei A.; Li T. Interplay between molecular conformation and intermolecular interactions in conformational polymorphism: A molecular perspective from electronic calculations of tolfenamic acid. Int. J. Pharm. 2011, 418, 179–186. 10.1016/j.ijpharm.2011.04.062. 21570454

[ref21] Surov A. O.; Szterner P.; Zielenkiewicz W.; Perlovich G. L. Thermodynamic and structural study of tolfenamic acid polymorphs. J. Pharm. Biomed. Anal. 2009, 50, 831–840. 10.1016/j.jpba.2009.06.045. 19632801

[ref22] Andersen K. V.; Larsen S.; Alhede B.; Gelting N.; Buchardt O. Characterization of Two Polymorphic Forms of Tolfenamic Acid, N-(2-Methyl-3-chlorophenyl)anthranilic Acid: Their Crystal Structures and Relative Stabilities. J. Chem. Soc., Perkin Trans. 2 1989, 10, 1143–1147. 10.1021/ac60303a028.

[ref23] Miglani Bhardwaj R.; Ho R.; Gui Y.; Brackemeyer P.; Schneider-Rauber G.; Nordstrom F. L.; Sheikh A. Y. Origins and Implications of Extraordinarily Soft Crystals in a Fixed-Dose Combination Hepatitis C Regimen. Cryst. Growth Des. 2022, 22 (7), 4250–4259. 10.1021/acs.cgd.2c00264.

[ref24] Hong R. S.; Miglani Bhardwaj R.; Henry R.; Mattei A.; Diwan M.; Thomas A.; Danzer G. D.; Sheikh A. Y. Distinct Hybrid Hydrates of Paritaprevir: Combined Experimental and Computational Assessment of their Hydration–Dehydration Behavior and Implications for Regulatory Controls. Cryst. Growth Des. 2021, 22 (1), 726–737. 10.1021/acs.cgd.1c01228.

[ref25] Sheikh A. Y.; Mattei A.; Miglani Bhardwaj R.; Hong R. S.; Abraham N. S.; Schneider-Rauber G.; Engstrom K. M.; Diwan M.; Henry R. F.; Gao Y.; et al. Implications of the Conformationally Flexible, Macrocyclic Structure of the First-Generation, Direct-Acting Anti-Viral Paritaprevir on Its Solid Form Complexity and Chameleonic Behavior. J. Am. Chem. Soc. 2021, 143 (42), 17479–17491. 10.1021/jacs.1c06837. 34637297

[ref26] Bauer J.; Spanton S.; Henry R.; Quick J.; Dziki W.; Porter W.; Morris J. Ritonavir: An extraordinary example of conformational polymorphism. Pharm. Res. 2001, 18 (6), 859–866. 10.1023/A:1011052932607. 11474792

[ref27] Bučar D.; Lancaster R. W.; Bernstein J. Disappearing polymorphs revisited. Angew. Chem., Int. Ed. 2015, 54 (24), 6972–6993. 10.1002/anie.201410356. PMC4479028 26031248

[ref28] Wang C.; Rosbottom I.; Turner T. D.; Laing S.; Maloney A. G. P.; Sheikh A. Y.; Docherty R.; Yin Q.; Roberts K. J. Molecular, Solid-State and Surface Structures of the Conformational Polymorphic Forms of Ritonavir in Relation to their Physicochemical Properties. Pharm. Res. 2021, 38, 971–990. 10.1007/s11095-021-03048-2. 34009625 PMC8217055

[ref29] Wang C.; Turner T. D.; Ma C. Y.; Pask C. M.; Rosbottom I.; Hong R.; Sheikh A.; Yin Q.; Roberts K. J. A Quaternary Solid-form of Ritonavir: an Oxalate Salt Oxalic Acid Co-crystal Acetone Solvate. CrystEngComm 2023, 25, 1782–1791. 10.1039/D2CE01612E.

[ref30] Yao X.; Henry R. F.; Zhang G. G. Z. Ritonavir Form III: A New Polymorph After 24 Years. J. Pharm. Sci. 2023, 112 (1), 237–242. 10.1016/j.xphs.2022.09.026. 36195132

[ref31] Parent S. D.; Smith P. A.; Purcell D. K.; Smith D. T.; Bogdanowich-Knipp S. J.; Bhavsar A. S.; Chan L. R.; Croom J. M.; Bauser H. C.; McCalip A.; et al. Ritonavir Form III: A Coincidental Concurrent Discovery. Cryst. Growth Des. 2023, 23 (1), 320–325. 10.1021/acs.cgd.2c01017.

[ref32] Chemburkar S. R.; Bauer J.; Deming K.; Spiwek H.; Patel K.; Morris J.; Henry R.; Spanton S.; Dziki W.; Porter W.; et al. Dealing with the impact of ritonavir polymorphs on the late stages of bulk drug process development. J. Am. Chem. Soc. 2000, 4 (5), 413–417. 10.1021/op000023y.

[ref33] Morissette S. L.; Soukasene S.; Levinson D.; Cima M. J.; Almarsson O. Elucidation of crystal form diversity of the HIV protease inhibitor ritonavir by high-throughput crystallization. Proc. Natl. Acad. Sci. U.S.A. 2003, 100 (5), 2180–2184. 10.1073/pnas.0437744100. 12604798 PMC151315

[ref34] Kawakami K.; Harada T.; Miura K.; Yoshihashi Y.; Yonemochi E.; Terada K.; Moriyama H. Relationship between crystallization tendencies during cooling from melt and isothermal storage: toward a general understanding of physical stability of pharmaceutical glasses. Mol. Pharmaceutics 2014, 11 (6), 1835–1843. 10.1021/mp400679m. 24731254

[ref35] Krämer K. Red–orange–yellow reclaims polymorph record with help from molecular cousin. 2020, https://www.chemistryworld.com/news/red-orange-yellow-reclaims-polymorph-record-with-help-from-molecular-cousin/4012160.article (accessed Jul 29, 2020).

[ref36] Tyler A. R.; Ragbirsingh R.; McMonagle C. J.; Waddell P. G.; Heaps S. E.; Steed J. W.; Thaw P.; Hall M. J.; Probert M. R. Encapsulated Nanodroplet Crystallization of Organic-Soluble Small Molecules. Chem 2020, 6 (7), 1755–1765. 10.1016/j.chempr.2020.04.009. 32685768 PMC7357602

[ref37] Schur E.; Bernstein J.; Price L. S.; Guo R.; Price S. L.; Lapidus S. H.; Stephens P. W. The (Current) Acridine Solid Form Landscape: Eight Polymorphs and a Hydrate. Cryst. Growth Des. 2019, 19 (8), 4884–4893. 10.1021/acs.cgd.9b00557.

[ref38] Serezhkin V. N.; Savchenkov A. V. Application of the Method of Molecular Voronoi–Dirichlet Polyhedra for Analysis of Noncovalent Interactions in Aripiprazole Polymorphs. Cryst. Growth Des. 2020, 20 (3), 1997–2003. 10.1021/acs.cgd.9b01645.

[ref39] Bobrovs R.; Seton L.; Dempster N. The reluctant polymorph: investigation into the effect of self-association on the solvent mediated phase transformation and nucleation of theophylline. CrystEngComm 2015, 17 (28), 5237–5251. 10.1039/c4ce02484b.

[ref40] Maher A.; Croker D. M.; Seaton C. C.; Rasmuson Å. C.; Hodnett B. K. Solution-mediated polymorphic transformation: Form II to Form III Piracetam in organic solvents. Cryst. Growth Des. 2014, 14 (8), 3967–3974. 10.1021/cg500565u.

[ref41] Rubin-Preminger J. M.; Bernstein J. 3-Aminobenzenesulfonic acid: a disappearing polymorph. Cryst. Growth Des. 2005, 5 (4), 1343–1349. 10.1021/cg049680y.

[ref42] Mullin J. W. Crystallization; Butterworth Heinemann, 2001; .

[ref43] Wang C.; Zhou L.; Zhang X.; Yang Y.; Yin Q.; Roberts K. J. The Role of Solvent Composition and Polymorph Surface Chemistry in the Solution-Mediated Phase Transformation Process of Cefaclor. Ind. Eng. Chem. Res. 2018, 57 (49), 16925–16933. 10.1021/acs.iecr.8b04462.

[ref44] Miller J. M.; Collman B. M.; Greene L. R.; Grant D. J.; Blackburn A. C. Identifying the stable polymorph early in the drug discovery–development process. Pharm. Dev. Technol. 2005, 10 (2), 291–297. 10.1081/pdt-200054467. 15926678

[ref45] Turner T. D.; Corzo D. M. C.; Toroz D.; Curtis A.; Dos Santos M. M.; Hammond R. B.; Lai X.; Roberts K. J. The influence of solution environment on the nucleation kinetics and crystallisability of para-aminobenzoic acid. Phys. Chem. Chem. Phys. 2016, 18 (39), 27507–27520. 10.1039/C6CP04320H. 27711471

[ref46] Toroz D.; Rosbottom I.; Turner T. D.; Corzo D. M. C.; Hammond R. B.; Lai X.; Roberts K. J. Towards an understanding of the nucleation of alpha-para amino benzoic acid from ethanolic solutions: a multi-scale approach. Faraday Discuss. 2015, 179, 79–114. 10.1039/C4FD00275J. 25920721

[ref47] Sullivan R. A.; Davey R. J.; Sadiq G.; Dent G.; Back K. R.; Ter Horst J. H.; Toroz D.; Hammond R. B. Revealing the roles of desolvation and molecular self-assembly in crystal nucleation from solution: benzoic and p-aminobenzoic acids. Cryst. Growth Des. 2014, 14 (5), 2689–2696. 10.1021/cg500441g.

[ref48] Rosbottom I.; Ma C. Y.; Turner T. D.; O’Connell R. A.; Loughrey J.; Sadiq G.; Davey R. J.; Roberts K. J. Influence of Solvent Composition on the Crystal Morphology and Structure of p-Aminobenzoic Acid Crystallized from Mixed Ethanol and Nitromethane Solutions. Cryst. Growth Des. 2017, 17 (8), 4151–4161. 10.1021/acs.cgd.7b00425.

[ref49] Hammond R. B.; Pencheva K.; Roberts K. J. An examination of polymorphic stability and molecular conformational flexibility as a function of crystal size associated with the nucleation and growth of benzophenone. Faraday Discuss. 2007, 136, 91–106. 10.1039/b616757h. 17955805

[ref50] Hammond R. B.; Pencheva K.; Roberts K. J. Structural variability within, and polymorphic stability of, nano-crystalline molecular clusters of L-glutamic acid and D-mannitol, modelled with respect to their size, shape and ‘crystallisability. CrystEngComm 2012, 14, 1069–1082. 10.1039/C1CE06174G.

[ref51] Davey R. J.; Allen K.; Blagden N.; Cross W. I.; Lieberman H. F.; Quayle M. J.; Righini S.; Seton L.; Tiddy G. J. T. Crystal engineering–nucleation, the key step. CrystEngComm 2002, 4 (47), 257–264. 10.1039/b203521a.

[ref52] Kashchiev D. Nucleation: Basic Theory with Applications; Butterworth Heinemann, 2000; .

[ref53] Du W.; Yin Q.; Gong J.; Bao Y.; Zhang X.; Sun X.; Ding S.; Xie C.; Zhang M.; Hao H. Effects of solvent on polymorph formation and nucleation of prasugrel hydrochloride. Cryst. Growth Des. 2014, 14 (9), 4519–4525. 10.1021/cg5006067.

[ref54] Davey R. J.; Schroeder S. L.; ter Horst J. H. Nucleation of organic crystals—a molecular perspective. Angew. Chem., Int. Ed. 2013, 52 (8), 2166–2179. 10.1002/anie.201204824. 23307268

[ref55] Davey R. J.; Dent G.; Mughal R. K.; Parveen S. Concerning the relationship between structural and growth synthons in crystal nucleation: solution and crystal chemistry of carboxylic acids as revealed through IR spectroscopy. Cryst. Growth Des. 2006, 6 (8), 1788–1796. 10.1021/cg060058a.

[ref56] Granberg R. A.; Ducreux C.; Gracin S.; Rasmuson Å. C. Primary nucleation of paracetamol in acetone–water mixtures. Chem. Eng. Sci. 2001, 56 (7), 2305–2313. 10.1016/S0009-2509(00)00439-5.

[ref57] Kulkarni S. A.; McGarrity E. S.; Meekes H.; ter Horst J. H. Isonicotinamide self-association: the link between solvent and polymorph nucleation. Chem. Commun. 2012, 48 (41), 4983–4985. 10.1039/c2cc18025a. 22498662

[ref58] Martin-Soladana P. M.; Diwan M.; Li H.; Nordstrom F. L.; Sheikh A. Y. Investigation of Nucleation under High-Shear Conditions for a Pharmaceutical Compound in an Unseeded System. Org. Process Res. Dev. 2019, 23, 2627–2636. 10.1021/acs.oprd.9b00155.

[ref59] Omar W.; Mohnicke M.; Ulrich J. Determination of the solid liquid interfacial energy and thereby the critical nucleus size of paracetamol in different solvents. Cryst. Res. Technol. 2006, 41 (4), 337–343. 10.1002/crat.200510584.

[ref60] Yang H.; Rasmuson Å. C. Nucleation of butyl paraben in different solvents. Cryst. Growth Des. 2013, 13 (10), 4226–4238. 10.1021/cg400177u.

[ref61] Khamar D.; Zeglinski J.; Mealey D.; Rasmuson Å. C. Investigating the Role of Solvent–Solute Interaction in Crystal Nucleation of Salicylic Acid from Organic Solvents. J. Am. Chem. Soc. 2014, 136 (33), 11664–11673. 10.1021/ja503131w. 25029039

[ref62] Mealey D.; Croker D. M.; Rasmuson Å. C. Crystal nucleation of salicylic acid in organic solvents. CrystEngComm 2015, 17 (21), 3961–3973. 10.1039/c4ce01428f.

[ref63] Zeglinski J.; Kuhs M.; Khamar D.; Hegarty A. C.; Devi R. K.; Rasmuson Å. C. Crystal Nucleation of Tolbutamide in Solution: Relationship to Solvent, Solute Conformation, and Solution Structure. Chem.—Euro. J. 2018, 24 (19), 4916–4926. 10.1002/chem.201705954. 29431236

[ref64] Rosbottom I.; Pickering J. H.; Etbon B.; Hammond R. B.; Roberts K. J. Examination of inequivalent wetting on the crystal habit surfaces of RS-ibuprofen using grid-based molecular modelling. Phys. Chem. Chem. Phys. 2018, 20, 11622–11633. 10.1039/c7cp08354h. 29662981

[ref65] Rosbottom I.; Pickering J. H.; Hammond R. B.; Roberts K. J. A Digital Workflow Supporting the Selection of Solvents for Optimizing the Crystallizability of p-Aminobenzoic Acid. Org. Process Res. Dev. 2020, 24, 500–507. 10.1021/acs.oprd.9b00261.

[ref66] Kaskiewicz P. L.; Rosbottom I.; Camacho Corzo D. M.; Hammond R. B.; Downie R.; Dowding P. J.; George N.; Roberts K. J. Influence of solution chemistry on the solubility, crystallisability and nucleation behaviour of eicosane in toluene:acetone mixed-solvents. CrystEngComm 2021, 23, 3109–3125. 10.1039/d1ce00322d.

[ref67] Miglani Bhardwaj R.; Ho R.; Gui Y.; Brackemeyer P.; Schneider-Rauber G.; Nordstrom F. L.; Sheikh A. Y. Origins and Implications of Extraordinarily Soft Crystals in a Fixed-Dose Combination Hepatitis C Regimen. Cryst. Growth Des. 2022, 22, 4250–4259. 10.1021/acs.cgd.2c00264.

[ref68] Jackson A. S. M.; Goberdhan D.; Dowding P. J.; Roberts K. J. Solution crystallisation of single and mixed n-alkanes, within the homologous series C16 to C23 from representative hydrocarbon fuel solvents. Fluid Phase Equilib. 2023, 570, 113705 10.1016/j.fluid.2022.113705. PMC10293999 37383827

[ref69] Mohd Noor S. Z.; Camacho D. M.; Yun Ma C.; Mahmud T. Effect of Crystallization Conditions on the Metastable Zone Width and Nucleation Kinetics of p-Aminobenzoic Acid in Ethanol. Chem. Eng. Technol. 2020, 43 (6), 1105–1114. 10.1002/ceat.201900679.

[ref70] Jiang S.; ter Horst J. H. Crystal Nucleation Rates from Probability Distributions of Induction Times. Cryst. Growth Des. 2011, 11, 256–261. 10.1021/cg101213q.

[ref71] Xiao Y.; Tang S. K.; Hao H.; Davey R. J.; Vetter T. Quantifying the Inherent Uncertainty Associated with Nucleation Rates Estimated from Induction Time Data Measured in Small Volumes. Cryst. Growth Des. 2017, 17, 2852–2863. 10.1021/acs.cgd.7b00372.

[ref72] Ma C. Y.; Geatches D.; Hsiao Y. W.; Kwokal A.; Roberts K. J. Role of Molecular, Crystal, and Surface Chemistry in Directing the Crystallization of Entacapone Polymorphs on the Au(111) Template Surface. Cryst. Growth Des. 2023, 23, 4522–4537. 10.1021/acs.cgd.3c00294. PMC10251417 37304395

[ref73] Rosbottom I.; Turner T. D.; Ma C. Y.; Hammond R. B.; Roberts K. J.; Yong C. W.; Todorov I. T. The structural pathway from its solvated molecular state to the solution crystallisation of the α- and β-polymorphic forms of *para* amino benzoic acid. Faraday Discuss. 2022, 235, 467–489. 10.1039/D1FD00112D. 35389403

[ref74] Rosbottom I.; Yong C. W.; Geatches D. L.; Hammond R. B.; Todorov I. T.; Roberts K. J. The integrated DL_POLY/DL_FIELD/DL_ANALYSER software platform for molecular dynamics simulations for exploration of the synthonic interactions in saturated benzoic acid/hexane solutions. Mol. Simul. 2021, 47 (2–3), 257–272. 10.1080/08927022.2018.1560441.

[ref75] Zou Z.; Beyerle E. R.; Tsai S.-T.; Tiwary P. Driving and characterizing nucleation of urea and glycine polymorphs in water. Proc. Natl. Acad. Sci. U.S.A. 2023, 120 (7), e2216099120 10.1073/pnas.2216099120. 36757888 PMC9963467

[ref76] Barron V. W.; Yong C. W.; Slowey A.; Todorov I. T.; Roberts K. J.; Hammond R. B. Comparison between the Intermolecular Interactions in the Liquid and Solid forms of Propellant 1,1,1,2 – Tetrafluoroethane. J. Mol. Liq. 2023, 383, 121993 10.1016/j.molliq.2023.121993.

[ref77] Toroz D.; Hammond R. B.; Roberts K. J.; Harris S.; Ridley T. Molecular dynamics simulations of organic crystal dissolution: The lifetime and stability of the polymorphic forms of para-amino benzoic acid in aqueous environment. J. Cryst. Growth 2014, 401, 38–43. 10.1016/j.jcrysgro.2014.01.064.

[ref78] Yong C. W. Descriptions and Implementations of DL_F Notation: A Natural Chemical Expression System of Atom Types for Molecular Simulations. J. Chem. Inf. Model. 2016, 56 (8), 1405–1409. 10.1021/acs.jcim.6b00323. 27455451

[ref79] Yong C. W.; Todorov I. T. DL_ANALYSER Notation for Atomic Interactions (DANAI): A Natural Annotation System for Molecular Interactions, Using Ethanoic Acid Liquid as a Test Case. Molecules 2017, 23 (1), 36 10.3390/molecules23010036. 29295538 PMC5943928

[ref80] Sacchi P.; Wright S. E.; Neoptolemou P.; Lampronti G. I.; Rajagopalan A. K.; Kras W.; Evans C. L.; Hodgkinson P.; Cruz-Cabeza A. J. Crystal size, shape, and conformational changes drive both the disappearance and reappearance of ritonavir polymorphs in the mill. Proc. Natl. Acad. Sci. U.S.A. 2024, 121 (15), e2319127121 10.1073/pnas.2319127121. 38557191 PMC11009673

[ref81] Moreira Pinheiro L. B.; Tao S.; Culbertson E.; Lima Barros de Araujo G.; Billinge S. J. L.; Ferreira F. F. Evaluation of the polymorphic forms of ritonavir and lopinavir in raw materials and co-milled systems. Int. J. Pharm. 2022, 628 (25), 122329 10.1016/j.ijpharm.2022.122329. 36280220 PMC9585847

[ref82] Hashemian S. M. R.; Sheida A.; Taghizadieh M.; Memar M. Y.; Hamblin M. R.; Bannazadeh Baghi H.; Sadri Nahand J.; Asemi Z.; Mirzaei H. Paxlovid (Nirmatrelvir/Ritonavir): A new approach to Covid-19 therapy?. Biomed. Pharmacother. 2023, 162, 114367 10.1016/j.biopha.2023.114367. 37018987 PMC9899776

[ref83] Chakraborty D.; Sengupta N.; Wales D. J. Conformational Energy Landscape of the Ritonavir Molecule. J. Phys. Chem. B 2016, 120 (19), 4331–4340. 10.1021/acs.jpcb.5b12272. 27123749

[ref84] Ilevbare G. A.; Liu H.; Edgar K. J.; Taylor L. S. Understanding Polymer Properties Important for Crystal Growth Inhibition-Impact of Chemically Diverse Polymers on Solution Crystal Growth of Ritonavir. Cryst. Growth Des. 2012, 12 (6), 3133–3143. 10.1021/cg300325p.

[ref85] Ilevbare G. A.; Liu H.; Edgar K. J.; Taylor L. S. Inhibition of solution crystal growth of ritonavir by cellulose polymers–factors influencing polymer effectiveness. CrystEngComm 2012, 14 (20), 6503–6514. 10.1039/c2ce25515d.

[ref86] Hong R. S.; Miglani Bhardwaj R.; Henry R.; Mattei A.; Diwan M.; Thomas A.; Danzer G. D.; Sheikh A. Y. Distinct Hybrid Hydrates of Paritaprevir: Combined Experimental and Computational Assessment of their Hydration–Dehydration Behavior and Implications for Regulatory Controls. Cryst. Growth Des. 2022, 22, 726–737. 10.1021/acs.cgd.1c01228.

[ref87] Ballard D. A.; Rosbottom I.; Roberts K. J. The crystallisation kinetics and particle characterisation of ritonavir from solution. University of Leeds 2017, (unpublished).

[ref88] Baird J. A.; Van Eerdenbrugh B.; Taylor L. S. A classification system to assess the crystallization tendency of organic molecules from undercooled melts. J. Pharm. Sci. 2010, 99 (9), 3787–3806. 10.1002/jps.22197. 20623696

[ref89] Law D.; Krill S. L.; Schmitt E. A.; Fort J. J.; Qiu Y.; Wang W.; Porter W. R. Physicochemical considerations in the preparation of amorphous ritonavir–poly (ethylene glycol) 8000 solid dispersions. J. Pharm. Sci. 2001, 90 (8), 1015–1025. 10.1002/jps.1054. 11536205

[ref90] Galek P. T.; Allen F. H.; Fábián L.; Feeder N. Knowledge-based H-bond prediction to aid experimental polymorph screening. CrystEngComm 2009, 11 (12), 2634–2639. 10.1039/b910882c.

[ref91] Technobis. Crystal16 unit. 2021, https://www.crystallizationsystems.com/products/crystal16/(accessed ).

[ref92] Camacho Corzo D. M.; Borissova A.; Hammond R. B.; Kashchiev D.; Roberts K. J.; Lewtas K.; More I. Nucleation mechanism and kinetics from the analysis of polythermal crystallisation data: methyl stearate from kerosene solutions. CrystEngComm 2014, 16 (6), 974–991. 10.1039/c3ce41098f.

[ref93] Liu P.; Kim B.; Friesner R. A.; Berne B. J. Replica exchange with solute tempering: A method for sampling biological systems in explicit water. Proc. Natl. Acad. Sci. U.S.A. 2005, 102 (39), 13749–13754. 10.1073/pnas.0506346102. 16172406 PMC1236566

[ref94] Lu C.; Wu C.; Ghoreishi D.; Chen W.; Wang L.; Damm W.; Ross G. A.; Dahlgren M. K.; Russell E.; Von Bargen C. D.; et al. OPLS4: Improving Force Field Accuracy on Challenging Regimes of Chemical Space. J. Chem. Theory Comput. 2021, 17 (7), 4291–4300. 10.1021/acs.jctc.1c00302. 34096718

[ref95] Schrödinger. Release 2023–3: Desmond Molecular Dynamics System, D. E. Shaw Research, New York, NY, 2021. Maestro-Desmond Interoperability Tools; Schrödinger: New York, NY, 2023.

[ref96] Schrödinger. Schrödinger Software Suite Release 2023–3; Schrödinger: New York, NY, 2023.

[ref97] Schrödinger. Schrödinger Release 2023–3: FEP+; Schrödinger, LLC: New York, NY, 2023.

[ref98] Ostwald W. Studies on the formation and transformation of solid compounds: Report I. Supersaturation and practicing cooling. Phys. Chem. 1897, 22U (1), 289–330. 10.1515/zpch-1897-2233.

[ref99] Hammond R. B.; Pencheva K.; Roberts K. J. Simulation of Energetic Stability of Facetted l-Glutamic Acid Nanocrystalline Clusters in Relation to Their Polymorphic Phase Stability as a Function of Crystal Size. J. Phys. Chem. B 2005, 109, 19550–19552. 10.1021/jp053546m. 16853527

[ref100] Stewart J. J. P. MOSOL MOPAC for Solid-State Physics. Quant. Chem. Prog. Exchange 1985, 5, 62–63.

[ref101] Augustyniak W.; Watson M. J.; Blundell C. D. Ritonavir Solution Structure and Relationship to Crystal Polymorphs; Butts C., Mari S., Eds.; NMR Conference: Baveno, Italy, 2015; p 147.SMASH 2015

[ref102] Cruz-Cabeza A. J.; Davey R. J.; Sachithananthan S. S.; Smith R.; Tang S. K.; Vetter T.; Xiao Y. Aromatic stacking–a key step in nucleation. Chem. Commun. 2017, 53 (56), 7905–7908. 10.1039/C7CC02423A. 28660260

[ref103] Kashchiev D.; Borissova A.; Hammond R. B.; Roberts K. J. Dependence of the Critical Undercooling for Crystallization on the Cooling Rate. J. Phys. Chem. B 2010, 114 (16), 5441–5446. 10.1021/jp100202m. 20369862

[ref104] Kashchiev D.; Borissova A.; Hammond R. B.; Roberts K. J. Effect of cooling rate on the critical undercooling for crystallization. J. Cryst. Growth 2010, 312 (5), 698–704. 10.1016/j.jcrysgro.2009.12.031. 20369862

