# Supplementary material for: Influence of Solvent Selection on the Crystallizability and Polymorphic Selectivity Associated with the Formation of the “Disappeared” Form I Polymorph of Ritonavir
Source: Mol Pharm. 2024 Jun 20;21(7):3525–39. doi: 10.1021/acs.molpharmaceut.4c00234 (PMC11220793; doi:10.1021/acs.molpharmaceut.4c00234)
Supplement: Supplementary file 1 — mp4c00234_si_001.pdf [file mp4c00234_si_001.pdf]

Supporting Information

Influence of Solvent Selection on the Crystallisability and Polymorphic Selectivity Associated with the Formation of the "Disappeared" Form I Polymorph of Ritonavir

Chang Wang[1,2], Cai Y. Ma[1], Richard S. Hong[3], Thomas D. Turner[1,$], Ian Rosbottom[1,#], Ahmad Y. Sheikh[3], Qiuxiang Yin[2], Kevin J. Roberts[1,]*

[1]Centre for the Digital Design of Drug Products, School of Chemical and Process Engineering, University of Leeds, Woodhouse Lane, Leeds, LS2 9JT, UK

[2]School of Chemical Engineering and Technology, State Key Laboratory of Chemical Engineering, Tianjin University, Tianjin 300072, China

[3]Molecular Profiling and Drug Delivery, Research and Development, AbbVie Inc., North Chicago, IL, 60064, USA

Current address:

[$]School of Chemistry, University of Leeds, Leeds LS2 9JT, UK

[#]GlaxoSmithKline, Gunnels Wood Rd, Stevenage, SG1 2NY, UK

* Corresponding Author: k.j.roberts@leeds.ac.uk

The supplementary material supports the main manuscript by providing further details of the following: Section S1 provides further details on induction time analysis using classical nucleation theory. **Figure S1** shows the DSC patterns of ritonavir nucleation in different solvents. **Figure S2** presents the exponential fitting of nucleation rates in toluene with supersaturation for estimating the nucleation rate at S = 5. **Table S1** lists the melting points, enthalpies of fusion, and full widths at half maximum (FWHM) for toluene, acetonitrile, ethyl acetate and acetone based on the DSC data. **Table S2** presents the comparisons of the relevant nucleation properties of ritonavir form I in different solvents and other compounds (butyl paraben [1], α-form PABA [2-4], methyl stearate [5], TFA form II [6]).

S1. Induction Time Analysis Using Classical Nucleation Theory

Measured induction times were analysed using classical nucleation theory (CNT) to calculate the nucleation rate (*J*) through the Arrhenius relationship as follows:

$$J = A\,exp\left(-\frac{\Delta G_c}{kT}\right) = A\,exp\left(-\frac{B}{ln^2 S}\right) \tag{1}$$

$$B = -\frac{16\pi\gamma^3 v^2}{3k^3T^3} \tag{2}$$

where A is the pre-exponential kinetic factor, B is the thermodynamic parameter, ΔG_c is the free energy assuming a spherical nucleus growth to critical size, S is the supersaturation ($S = C/C^*$; C = concentration in solution (g/g solvent), C^* = solubility at the crystallisation temperature), k is the Boltzmann constant, T is the nucleation absolute temperature, γ is the effective interfacial energy, and v is the molecule volume.

The pre-exponential kinetic factor, *A*, associating with the molecular kinetics of the nucleation process, can be determined [7] by Eq. (3):

$$A = z f^* C_0 \tag{3}$$

where C_0 is the concentration of nucleation sites, f^* is the attachment frequency of building units to a nucleus and can be impacted by changes of solvent, supersaturation and molecular conformation of the solute, z is the Zeldovich factor which can be derived and calculated [8, 9] by:

$$z = \frac{ln^2 S}{\sqrt{12\pi B}} \tag{4}$$

Considering the dependence of Zeldovich factor and attachment frequency on supersaturation, and the higher concentration leading to higher attachment frequency, the pre-exponential kinetic factor, *A*, and the classical nucleation expression can be re-written as Eqs. (5) and (6), respectively:

$$A = A_0\, S\, ln^2 S \tag{5}$$

$$J = A_0 \, S \, ln^2 S \, \exp\left(\frac{B}{ln^2 S}\right) \tag{6}$$

where the pre-exponential kinetic factor constant, A_0, and the thermodynamic parameter B can be derived from the intercept and slope, respectively, by plotting the linear function of $ln \left(\frac{J}{S \, ln^2 S}\right)$ versus $\frac{1}{in^2 S}$.

The attachment rate, $f^* C_0$, can be estimated by combining equations (3) – (5):

$$f^* C_0 = A_0 S \sqrt{12 \pi B} \tag{6}$$

From the CNT, the critical size, r_c, for the nucleation cluster was calculated from:

$$r_c = \frac{2v\gamma}{kTlnS} \tag{7}$$

S2. DSC Pattern and Data Analysis

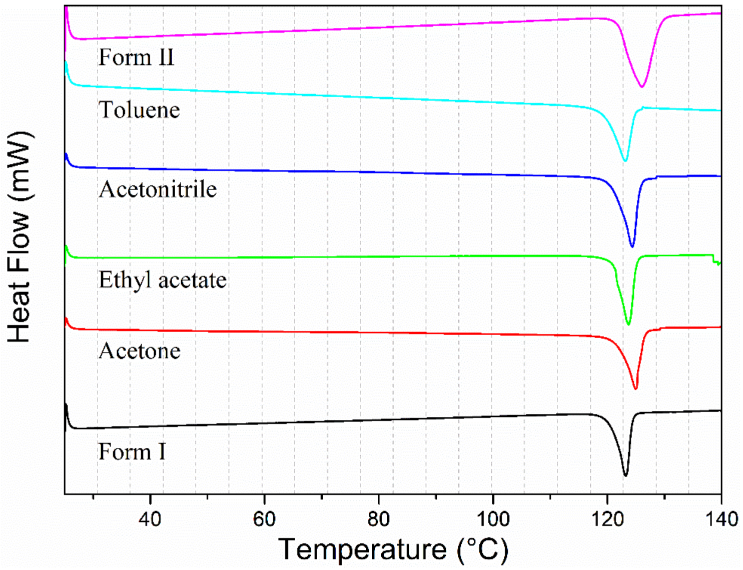

Figure S1. The DSC patterns of ritonavir nucleation in different solvents.

Table S1. Melting points, enthalpies of fusion, and FWHMs for toluene, acetonitrile, ethyl acetate and acetone based on the DSC data. Note that unfortunately, the original DSC data for ethyl acetate is no longer available due to an instrument software upgrade.

Solvent	Melting point (^{o}C)	Enthalpy of fusion (kJ mol^{-1})	Half height width (^{o}C)
Toluene	123.00	-38.43	3.45
Acetonitrile	124.14	-40.19	2.33
Ethyl acetate	N/A	N/A	N/A
Acetone	125.00	-41.10	2.21

S3. Fitting of Nucleation Rate in Toluene with Supersaturation

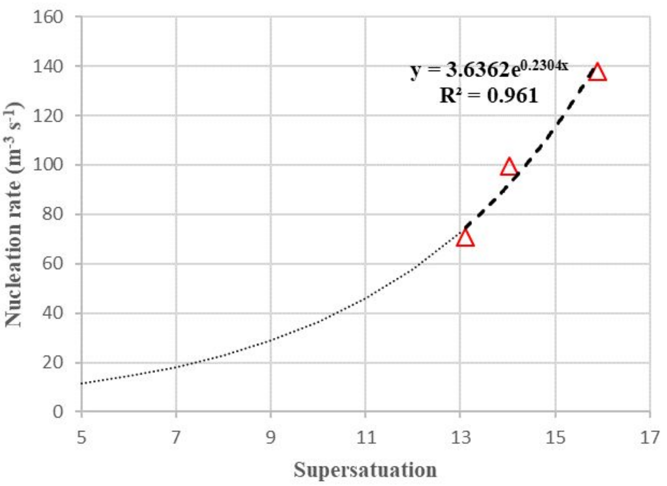

Figure S2. Exponential fitting of nucleation rates in toluene with supersaturation for estimating the nucleation rate of 11.51 m^{-3} s^{-1} at S = 5.

S4. Comparisons of Nucleation Properties of Different Compounds

Table S2 compares the relevant nucleation properties of ritonavir form I in different solvents and other compounds (butyl paraben [1], α-form PABA [2-4], methyl stearate [5], TFA form II [6]) from literature. The nucleation rate, critical nuclei size, number of nuclei and effective surface energy obtained from this study are generally compatible with these compounds from literature with two compounds (methyl stearate and TFA form II) having much higher nucleation rates obtained using the KBHR method [10, 11] with progressive nucleation mechanism. The supersaturation values for nucleation studies of ritonavir form I in various solvents in the current study are serval times higher than the other compounds from literature with the nucleation driving force being also higher than butyl paraben, and the average induction time being in the similar order of magnitude to α-form PABA but about 10 times longer than butyl paraben.

Table S2. Nucleation parameters and the associated properties of different compounds in various solvents. Note that the grey filled area indicates the corresponding solvent not being studied in the literature, the symbol (-) means the value of the property not being available and the grey area presents the solvent not being studied in the paper.

Property	Solvent	**Butyl paraben** [1] CNT, isothermal	**α-PABA** [a, b] [a= [2, 3]; b= [4]] CNT, isothermal	**Ritonavir form I** (this study) CNT, isothermal	**Methyl stearate** [5] KBHR-PN, polythermal	**TFA form II** [6] KBHR-PN, polythermal
J (m^{-3} s^{-1})	Acetone	3076.92-4000.00		58.17 - 245.79		
	Acetonitrile		8 - 662 [a]	62.79 - 659.70		1.08×10^{13} – 2.48×10^{13}
	Ethanol	147.06 - 1418.44	86.58 - 177.97 [b]			7.50×10^{8} – 9.74×10^{9}
	Ethyl acetate	340.72 - 1801.80	4 - 346 [a]	64.27 - 89.43		
	Toluene			70.95 - 138.07	4.56×10^{22} – 1.04×10^{23}	4.77×10^{10} – 6.39×10^{10}
r_c (Å)	Acetone	4 - 20		9.59 - 10.93		
	Acetonitrile		4 - 68 [a]	12.88 - 14.20		5.2 - 11.1
	Ethanol	8 - 16	4.8 - 19.8 [b]			5.5 - 12.5
	Ethyl acetate	8 - 16	9 - 60 [a]	5.88 - 6.26		
	Toluene			6.81 - 7.32	7.3 – 11.7	5.6 - 11.1
i_c (#)	Acetone	1 - 123		4.08 - 6.06		
	Acetonitrile		1.61 - 7908.55 [a]	9.91 - 13.27		1.97 - 19.17
	Ethanol	7 - 64	3 - 195 [b]			2.33 - 27.37
	Ethyl acetate	7 - 63	18.34 - 5432.80 [a]	0.94-1.13		
	Toluene			1.47 - 1.82	3 – 14	2.43 - 19.17
γ (mJ m^{-2})	Acetone	0.3		3.39		
	Acetonitrile		1.33 [a]	5.04		1.64 - 2.73
	Ethanol	1.13	0.85 - 1.31 [b]			4.27 - 5.74
	Ethyl acetate	1.13	1.96 [a]	2.12		
	Toluene			4.08	1.18 – 1.88	2.83 - 3.93
S	Acetone	1.04 - 1.10		4.20 - 5.14		
	Acetonitrile		1.08 - 1.20 [a]	5.16 - 6.10		1.29 - 1.50

	Ethanol	1.10 - 1.22	1.07 - 1.15 [b]			2.05 - 2.99
	Ethyl acetate	1.11 - 1.22	1.12 - 1.24 [a]	4.80 - 5.31		
	Toluene			13.11 - 15.89	1.49 – 1.58	1.71 - 1.81
t_{ind}	Acetone	50 - 6861		5343 - 17192		
	Acetonitrile		4500 - 28000 [a]	1515 - 15925		-
	Ethanol	141 - 3172	3746 - 7700 [b]			-
	Ethyl acetate	111 - 1360	7500 - 29500 [a]	11182 - 15559		
	Toluene			5343 - 14094	-	-
RTlnS (J mol^{-1})	Acetone	94 - 224		3378 - 3854		
	Acetonitrile		-	3863 - 4257		-
	Ethanol	284 - 471	-			-
	Ethyl acetate	227 - 468	-	3693 - 3930		
	Toluene			6058 - 6511	-	-
**Mole fraction solubility**	Acetone	0.31		0.0043		
	Acetonitrile		0.060 [a]	0.00066		0.0005 - 0.001
	Ethanol	0.26	-			0.004 - 0.0066
	Ethyl acetate	0.25	0.076 [a]	0.00133		
	Toluene			0.00022	0.03 – 0.06	0.0008 - 0.0015
**Viscosity** (mPa s)	Acetone	0.33		0.33		
	Acetonitrile		-	0.38		0.25 - 0.64
	Ethanol	1.08	-			0.72 - 1.95
	Ethyl acetate	0.46	-	0.46		
	Toluene			0.59	-	0.45 - 0.75

References

(1) Yang, H.; Rasmuson, Å. C. Nucleation of butyl paraben in different solvents. *Crystal Growth & Design* **2013**, *13* (10), 4226-4238.

(2) Sullivan, R. A. Molecules, Clusters and Crystals: The Crystallisation of p-aminobenzoic acid from Solution. University of Manchester, 2015.

(3) Sullivan, R. A.; Davey, R. J.; Sadiq, G.; Dent, G.; Back, K. R.; Ter Horst, J. H.; Toroz, D.; Hammond, R. B. Revealing the roles of desolvation and molecular self-assembly in crystal nucleation from solution: benzoic and p-aminobenzoic acids. *Crystal Growth & Design* **2014**, *14* (5), 2689-2696.

(4) Toroz, D.; Rosbottom, I.; Turner, T. D.; Corzo, D. M. C.; Hammond, R. B.; Lai, X.; Roberts, K. J. Towards an understanding of the nucleation of alpha-para amino benzoic acid from ethanolic solutions: a multi-scale approach. *Faraday Discuss.* **2015**, *179*, 79-114.

(5) Corzo, D. M. C.; Roberts, K. J.; More, I.; Lewtas, K. Solubility and Nucleation of Methyl Stearate as a Function of Crystallization Environment. *Energy Fuels* **2018**, *32* (3), 3447–3459.

(6) Liu, Y.; Ma, C. Y.; Gong, J.; Roberts, K. J. The Influence of Solvent Selection upon the Crystallisability and Nucleation Kinetics Tolfenamic Acid Form II. *Crystal Growth & Design* **2023**, *23* (8), 5846–5859.

(7) Kashchiev, D. *Nucleation: Basic Theory with Applications*; Butterworth Heinemann, 2000.

(8) Dey, D.; Thomas, S. P.; Spackman, M. A.; Chopra, D. 'Quasi isostructural polymorphism' in molecular crystals: inputs from interaction hierarchy and energy frameworks. *Chemical Communications* **2016**, *52* (10), 2141-2144.

(9) Qu, H.; Louhi-Kultanen, M.; Rantanen, J.; Kallas, J. Solvent mediated phase transformation kinetics of an anhydrate/hydrate system. *Crystal Growth & Design* **2006**, *6* (9), 2053-2060.

(10) Kashchiev, D.; Borissova, A.; Hammond, R. B.; Roberts, K. J. Dependence of the Critical

Undercooling for Crystallization on the Cooling Rate. *J. Phys. Chem. B* **2010**, *114* (16), 5441–5446.

(11) Kashchiev, D.; Borissova, A.; Hammond, R. B.; Roberts, K. J. Effect of cooling rate on the critical undercooling for crystallization. *Journal of Crystal Growth* **2010**, *312* (5), 698-704.
